# Genome, HLA and polygenic risk score analyses for prevalent and persistent cervical human papillomavirus (HPV) infections

**DOI:** 10.1038/s41431-023-01521-7

**Published:** 2024-01-10

**Authors:** Sally N. Adebamowo, Adebowale Adeyemo, Amos Adebayo, Peter Achara, Bunmi Alabi, Rasheed A. Bakare, Ayotunde O. Famooto, Kayode Obende, Richard Offiong, Olayinka Olaniyan, Sanni Ologun, Charles Rotimi, ACCME Research Group as part of the H3Africa Consortium, Saurayya S. Abdullahi, Maryam Abdulsalam, Ruxton Adebiyi, Victor Adekanmbi, Bukunmi Adelekun, Segun Adeyemo, Gerald Akabueze, Bernice Akpobome, Stella Akpomiemie, Gabriel O. Alabi, Chinyere Anichebe, Claire Anyanwu, Miriam C. Ayogu, Dorcas J. Bako, Patience Bamisaiye, Nkechi U. Blessing, Osa A. Chinye, Patrick Dakum, Eileen Dareng, Grace Dwana, Juliet I. Erhunmwonsere, Emelda O. Eze, Tolani A. Fagbohun, Temitope Filade, Toluwalope Gbolahan, Gloria C. Anaedobe, Stella Ibezim, Racheal Iwaloye, Jesse James, Dayo Kehinde, Fiyinfoluwa Makinde, Jessica Mase, Charles Mensah, Florence A. Nwoko, Kayode Obende, George Odonye, Folake Odubore, Funmi Odunyemi, Michael Odutola, Uzoamaka Oguama, Tochukwu Oguoma, Temitayo Oladimeji, Toyosi Olawande, Temitope Olukomogbon, Sefunmi Oluwole, Gladys Omenuko, Nkiruka Onwuka, Yinka Owoade, Thelma C. Ugorji, Syntyche Yohanna, Ibrahim Yusuf, Clement A. Adebamowo

**Affiliations:** 1 Department of Epidemiology and Public Health, University of Maryland School of Medicine, Baltimore, MD 21201 USA; 2 Greenebaum Comprehensive Cancer Center, University of Maryland School of Medicine, Baltimore, MD 21201 USA; 3 National Human Genome Research Institute, Bethesda, MD USA; 4 Asokoro District Hospital, Abuja, Nigeria; 5 Federal Medical Center, Keffi, Nigeria; 6 Wuse General Hospital, Abuja, Nigeria; 7 Department of Microbiology, University College Hospital, University of Ibadan, Ibadan, Nigeria; 8 Institute of Human Virology Nigeria, Abuja, Nigeria; 9 Garki Hospital Abuja, Abuja, Nigeria; 10 University of Abuja Teaching Hospital, Gwagwalada, Abuja, Nigeria; 11 National Hospital Abuja, Abuja, Nigeria; 12 Kubwa General Hospital, Abuja, Nigeria; 13 Center for Bioethics and Research, Ibadan, Nigeria; 14 Department of Public Health and Primary Care, Centre for Cancer Genetic Epidemiology, University of Cambridge, Cambridge, UK; 15 Centre for Big Data Research in Health, University of New South Wales, Sydney, NSW Australia

**Keywords:** Genetic markers, Genetics research, Cervical cancer

## Abstract

Genetic variants that underlie susceptibility to cervical high-risk human papillomavirus (hrHPV) infections are largely unknown. We conducted discovery genome-wide association studies (GWAS), replication, meta-analysis and colocalization, generated polygenic risk scores (PRS) and examined the association of classical HLA alleles and cervical hrHPV infections in a cohort of over 10,000 women. We identified genome-wide significant variants for prevalent hrHPV around LDB2 and for persistent hrHPV near TPTE2, SMAD2, and CDH12, which code for proteins that are significantly expressed in the human endocervix. Genetic variants associated with persistent hrHPV are in genes enriched for the antigen processing and presentation gene set. HLA-DRB1*13:02, HLA-DQB1*05:02 and HLA-DRB1*03:01 were associated with increased risk, and HLA-DRB1*15:03 was associated with decreased risk of persistent hrHPV. The analyses of peptide binding predictions showed that HLA-DRB1 alleles that were positively associated with persistent hrHPV showed weaker binding with peptides derived from hrHPV proteins and vice versa. The PRS for persistent hrHPV with the best model fit, had a P-value threshold (PT) of 0.001 and a p-value of 0.06 (-log10(0.06) = 1.22). The findings of this study expand our understanding of genetic risk factors for hrHPV infection and persistence and highlight the roles of MHC class II molecules in hrHPV infection.

## Introduction

Human papillomavirus (HPV) infection is the second most common oncogenic infection in the world, accounting for 31.4% of all infection-attributable cancers (690,000 of 2.2 million cases) globally [1, 2]. Persistent HPV infection (which is HPV test positivity at consecutive timepoints, usually several months apart) is a necessary but not sufficient cause of anogenital and oropharyngeal cancers [3]. The lifetime prevalence of HPV infection in women is over 80%. Most infections occur shortly after sexual debut, and over 90% are cleared within 2 years. Environmental factors, including smoking, long-term hormonal contraceptive use, HIV coinfection and the vaginal microenvironment (coinfections, anti-inflammatory cytokines and cervicovaginal microbiota), affect the risks of persistence and clearance of HPV infections.

Several observations suggest a role for genetic factors in HPV prevalence (which is HPV test positivity at a single timepoint only, usually at baseline) and persistence, but this is largely understudied. Previously, we conducted the first genome-wide association study (GWAS) of HPV infection and identified suggestive candidate risk loci for prevalent and persistent cervical HPV infection [4]. The top three variants associated with prevalent infection were clustered in Krüppel-like Factor 12 gene (*KLF12)*, while those associated with persistent infection were near Death Associated Protein gene (*DAP*), Catenin Delta 2 (*CTNND2*), MicroRNA 365b gene *(MIR365-2)* and Nuclear Receptor Subfamily 5 Group A Member 2 gene *(NR5A2)*. To date, no other GWAS of cervical HPV infection has been conducted. Candidate gene association studies provide additional insights into the genetic risks of prevalent and persistent cervical HPV infections, but few have been conducted in African populations. These studies reported associations between SNPs in deoxyuridine triphosphatase (*DUT*), peroxiredoxin 3 (*PRDX3*), ribosomal protein S19 (*RPS19*), general transcription factor IIH subunit 4 (*GTF2H4*), 2’-5’-oligoadenylate synthetase 3 (*OAS3*) and sulfatase 1 (*SULF1*) gene regions and HPV persistence [5–7].

The human leukocyte antigen (HLA) genes located in the major histocompatibility complex (MHC) region play major roles in the immune response to infections by encoding HLA proteins specialized to present antigenic peptides to T-cell receptors (TCRs) [8]. They play major roles in adaptive immunity, including facilitating the clearance of HPV-infected cells. Polymorphisms in HLA alleles may cause the production of proteins that have lower binding affinity to HPV antigens and are less likely to recognize cervical HPV infections, thus causing a higher likelihood of persistent infections [9]. Because the genetic structure of the MHC region is highly complex and diverse among populations, population-specific studies of associations between HLA alleles and diseases are warranted [10]. There have been few studies of HLA alleles, haplotypes, and amino acids and cervical HPV infections, particularly in Africa. The few African studies reported that DRB1*, DQB1* and DQA1* were associated with HPV infections. In this study, we conducted comprehensive genome-wide and HLA allele studies of the risks of prevalent and persistent cervical high-risk (hr) HPV infections in self-reported HIV-negative women.

## Results

### Data

The African Collaborative Center for Microbiome and Genomics Research (ACCME) cohort profile and characteristics of the study participants have been described in detail elsewhere [11]. The mean (standard deviation) age of the participants was 38 [10] years. The PC plots of the genotypes showed clustering of the study participants by ancestry (Supplementary Fig. 1).

### Genome-wide association study

A total of 903 women with hrHPV infections passed the filters and controls for the case-control GWAS, of which 224 had hrHPV infections at baseline only and were included in the prevalent hrHPV GWAS, and 679 had hrHPV infections at baseline and follow-up, and were included in the persistent hrHPV GWAS. Also, 9,846 HPV-negative women passed the filters and controls and were included as controls in the GWAS.

The Miami plot, Supplementary Fig. 2, shows all the SNPs associated with both prevalent and persistent hrHPV infections. Supplementary Table 1 shows the top SNPs associated with prevalent hrHPV infections. One variant, rs116471799, located on chromosome 4, was significantly associated with prevalent hrHPV (odds ratio [OR], *p*-value [*p*]: 3.63, *p* = 1.76 × 10^-8^). This variant is located 77 kb 3’ of LIM domain binding 2 (*LDB2*). Over 200 SNPs reached borderline genome-wide significance for association with prevalent hrHPV. Supplementary Table 1 also shows the top SNPs associated with persistent hrHPV infections, four of which reached genome-wide significance. The SNP with the strongest significant association, rs2342234 (OR: 0.35, *p* = 1.50 × 10^−8^), is located 37 kb 3’ of Transmembrane Phosphoinositide 3-Phosphatase and Tensin Homolog 2 (*TPTE2*), a protein coding gene. The second significantly associated SNP, rs115537401 (OR: 2.50, *p* = 3.26 × 10^−8^), is located 30 kD of SMAD Family Member 2 (*SMAD2*). The third and fourth significantly associated SNPs, rs1879062 (OR: 1.79, *p* = 3.81 × 10^−8^) and rs1028206 (OR: 1.78, *p* = 4.45 × 10^−8^), were clustered on chromosome 5 (*D’* = 1, *r*^*2*^ = 0.97). Over 200 SNPs reached borderline genome-wide significance for persistent hrHPV. Supplementary Fig. 3 shows the top SNPs associated with hrHPV in the multinomial logistic regression analysis.

### Replication studies

To evaluate the replication of the top variants in the discovery study, we examined them in the Prospective Abuja Cohort Study (PACS), the only other available GWAS of hrHPV [4], to our knowledge. We were able to replicate rs116054643 for the association with prevalent hrHPV (discovery cohort - OR: 1.88, *p* = 4.52 × 10^−7^; replication cohort - OR: 1.68, *p* = 0.01, Supplementary Table 1) and rs12448674 for the association with persistent hrHPV (discovery cohort - OR: 0.75, *p* = 4.18 × 10^−7^; replication cohort - OR: 0.71, *p* = 0.04, Supplementary Table 1). For the top variants associated with prevalent and persistent hrHPV in the discovery cohort, we observed that the MAF was similar in the replication cohort, but the direction and magnitude of the association differed for approximately half of the variants. None of the tops SNPs in the present study were found in published cervical cancer GWAS from the NHGRI-EBI GWAS catalog, probably because of differences in the phenotypes (HPV infection versus cervical cancer) and populations studied (those studies did not include African populations while this study is exclusively of Africans).

We investigated the transferability of previously reported SNPs associated with hrHPV and cervical cancer in the ACCME study sample. Of the 339 SNPs, 254 were present in our dataset. Six SNPs (or 2%, 6/254) showed exact replication, i.e., consistent direction of effect for the alleles and *p* < 0.05, for prevalent hrHPV, and eighteen SNPs (or 7%, 18/254) showed exact replication for association with persistent hrHPV (Supplementary Table 2).

### Meta-analysis

**Fig. 1:**
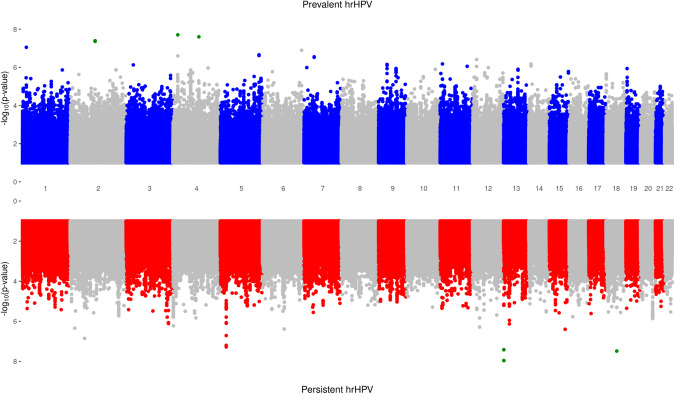
Miami plot for meta-analysis of ACCME and PACS cohorts for prevalent and persistent high-risk HPV.

We conducted a meta-analysis with the GWAS of ACCME and PACS using METAL and METASOFT software. The meta-analysis yielded genome-wide significant loci *LDB2* (rs116471799, *p* = 1.98 × 10^−8^), Protein Phosphatase 3 Catalytic Subunit Alpha (*PPP3CA)* (rs116054643, *p* = 2.50 × 10^−8^) and NCK Adaptor Protein 2 (*NCK2)* (rs138289957, *p* = 4.03 × 10^−8^; rs13407090, *p* = 4.12 × 10^−8^; rs75543399, *p* = 4.34 × 10^−8^) associated with the prevalent hrHPV (Table 1). The meta-analysis yielded genome-wide significant loci, *TPTE2* (rs2342234, *p* = 1.10 × 10^−8^ and rs2152687, *p* = 3.80 × 10^−8^), associated with persistent hrHPV (Table 1). Figure 1 shows a Miami plot for the prevalent and persistent hrHPV meta-analyses. The meta-analysis results from METAL and Metasoft were similar. The regional plots for the top variants in the meta-analyses are shown in Supplementary Fig. 4.

**Table 1:** Meta-analysis results for Top 19 SNPs Associated with Baseline Prevalent and Persistent Cervical hrHPV Infections.

SNP	Chr	Base Position	Near gene	Ref allele	Direction	*N*	SE	*P*-value
Prevalent hrHPV								
rs116471799	4	16424409	*LDB2*	C	**+ +**	10815	5.614	1.98 × 10^−8^
rs116054643	4	101300926	*PPP3CA*	T	**+ +**	10815	5.573	2.50 × 10^−8^
rs138289957	2	105685243	*NCK2*	A	**+ +**	10815	5.489	4.03 × 10^−8^
rs13407090	2	105690494	*NCK2*	A	**+ +**	10815	5.486	4.12 × 10^−8^
rs75543399	2	105670030	*NCK2*	A	**+ +**	10815	5.476	4.34 × 10^−8^
rs4654837	1	23233507	*HTR1D*	T	**+ +**	10815	5.348	8.88 × 10^−8^
rs73784569	6	163291426	*PACRG*	T	**+ +**	10815	5.282	1.28 × 10^−7^
rs144361576	5	166842323	*TENM2*	A	**+ +**	10815	5.181	2.21 × 10^−7^
rs75713170	5	166840858	*TENM2*	T	**- -**	10815	−5.165	2.40 × 10^−7^
rs149580050	4	16517334	*LDB2*	C	**- -**	10815	−5.157	2.50 × 10^−7^
Persistent hrHPV								
rs2342234	13	19385993	*TPTE2*	T	+ +	11195	5.71	1.10 × 10^−8^
rs2152687	13	19382475	*TPTE2*	C	+ +	11195	5.50	3.80 × 10^−8^
rs1879062	5	23044423	*CDH12*	A	- -	11195	−5.44	5.24 × 10^−8^
rs1028206	5	23046022	*CDH12*	A	- -	11195	−5.41	6.30 × 10^−8^
rs10180404	2	54218617	*TSPYL6*	T	+ +	11195	5.26	1.41 × 10^−7^
rs72750684	5	23050313	*CDH12*	A	+ +	11195	5.20	1.94 × 10^−7^
rs2180716	6	87974113	*SPACA1*	A	+ -	11195	5.06	4.13 × 10^−7^
rs7599455	2	15328667	*NBAS*	A	+ +	11195	5.04	4.58 × 10^−7^
rs7137933	12	26616574	*ITPR2*	A	+ +	11195	5.02	5.12 × 10^−7^
rs112783887	4	1355653	*UVSSA*	T	+ +	11195	4.99	5.94 × 10^−7^

### Functional annotation

Functional annotation in the HaploReg database revealed that rs116471799 (77 kb 3’ of *LDB2*), which was associated with prevalent hrHPV at the genome-wide significance level, is associated with altering 17 regulatory motifs. The top variants associated with persistent hrHPV at the genome-wide significance level were associated with altering regulatory motifs and showed both promoter and enhancer activities of the histone modification signatures H3K4me1, H3K4me3, H3K27ac and H3K9ac in several tissues. rs2342234 (37 kb 3’ of *TPTE2*) is associated with H3K9ac promoter histone marks in H1 Bone Morphogenetic Protein 4 (BMP4)-derived trophoblast cultured cells. rs115537401 (30 kb 3’ of *SMAD2)* is associated with H3K9ac and H3K4me3 promoter histone marks in primary mononuclear cells from peripheral blood and lungs, respectively, and H3K4me1 enhancer histone marks in H1 BMP4-derived trophoblast cultured cells, mesenchymal stem cell-derived adipocyte cultured cells, breast myoepithelial primary cells, and several other tissues.

Querying the Genotype-Tissue Expression (GTEx) database, we found that *LDB2* is highly expressed in the uterus (median transcript per million [TPM] = 84.6, endocervix (median TPM = 61.4) and subcutaneous adipose tissue (median TPM = 54.4) (Supplementary Fig. 5). *TPTE2* is mainly expressed in the testes (median TPM = 18) and in EBV-transformed lymphocytes (median TPM ~ 1). Its median TPM in other tissues was <1 (Supplementary Fig. 6). *SMAD2* is expressed in the uterus (median TPM = 6.3) and endocervix (median TPM 5.8) (Supplementary Fig. 7).

### Colocalization analysis

For the colocalization analysis of the top variants identified in the meta-analysis for prevalent hrHPV and GTEX data, 5 SNPs were identified in the vagina eQTLs for the variants on chromosome 2 (rs138289957, rs13407090, rs75543399), the posterior probability [pp] of a shared variant was 6.44%). For variants on chromosome 4, 8 SNPs were identified in the vagina eQTLs for rs116471799 (pp of a shared variant was 2.97%); 30 SNPs were identified in the vagina eQTLs (pp of a shared variant was 9.83%) and 36 SNPs in the uterus eQTLs for rs116054643 (pp of a shared variant was 6.27%); for rs116054643. For persistent hrHPV, 13 SNPs had significant eQTLs for the uterus and were present in the meta-analysis within 500 Kbs of rs2342234 and rs2152687 on chromosome 13 (Supplementary Table 3).

### Classic HLA associations

The most common HLA alleles in the study population were DPB1*01.01, DQA1*01:02 and DQB1*06:02. The frequency of DPB1*01.01 was 0.42 for the total population and in hrHPV negative women, 0.44 in women with prevalent and persistent hrHPV infections. The frequency of DQA1*01:02 was 0.38 in the total population, hrHPV negative women and women with prevalent hrHPV infection, and 0.37 in women with persistent hrHPV infection. The frequency of DQB1*06:02 was 0.27, in the total population, hrHPV negative women and women with prevalent hrHPV infection, and 0.24 in women with persistent hrHPV infection.

None of the HLA alleles, haplotypes or amino acids were significantly associated with prevalent hrHPV. In adjusted models, four HLA alleles, DRB1*15:03, DRB1*13:02, DQB1*05:02 and DRB1*03:01, were significantly associated with persistent hrHPV in the ACCME cohort; DRB1*03:01 and DQB1*02:01 remained borderline significant in the PACS cohort; and DRB1*15:03, DRB1*13:02, DRB1*03:01 and DQB1*06:02, were significantly associated with persistent hrHPV in the combined cohort analysis (Table 2). Most of these significant alleles are included in the haplotypes associated with persistent hrHPV, including C*07:01 - DQB1*06:02, B*58:02 - DRB1*15:03 and DQB1*05:02 - DRB1*13:02. (Supplementary Table 4). A total of 37 haplotypes were significantly associated with persistent hrHPV, and 46% (17/37) of them were located on DRB1. Eighty-five amino acids were significantly associated with persistent hrHPV. Approximately 32% (27/85) of the significant amino acid positions were located on DRB1. DRB1_42_Serine (OR: 1.45, *p* = 8.19 × 10^−05^) and DRB1_59_Tyrosine (OR: 1.54, *p* = 2.27 × 10^−04^) showed the strongest associations (Supplementary Table 5) with persistent hrHPV.

**Table 2:** HLA Alleles Associated with Persistent Cervical hrHPV Infection.

Allele	Frequency Cases	Frequency Controls	Odds Ratio (95% CI)	*P-*value	FDR-Adjusted *P*-value
Discovery in ACCME Cohort					
DRB1*15:03	0.161	0.199	0.77 (0.66–0.89)	7.20 × 10^−04^	0.006
DRB1*13:02	0.074	0.054	1.45 (1.17–1.79)	5.85 × 10^−04^	0.006
DQB1*05:02	0.056	0.039	1.48 (1.17–1.89)	0.001	0.015
DRB1*03:01	0.099	0.076	1.29 (1.07–1.56)	0.007	0.041
DQB1*06:02	0.241	0.275	0.84 (0.74–0.96)	0.012	0.068
DQB1*02:01	0.142	0.119	1.21 (1.03–1.43)	0.017	0.068
DQB1*02:02	0.050	0.066	0.76 (0.59–0.98)	0.037	0.111
Replication in PACS Cohort					
DRB1*15:03	0.156	0.184	0.84 (0.48–1.46)	0.543	0.611
DRB1*13:02	0.107	0.059	2.35 (1.07–5.16)	0.031	0.142
DQB1*05:02	-	-	-	-	-
DRB1*03:01	0.156	0.071	2.37 (1.21–4.65)	0.011	0.094
DQB1*06:02	0.264	0.263	0.97 (0.60–1.55)	0.909	0.909
DQB1*02:01	0.142	0.119	2.10 (1.08–4.09)	0.020	0.061
DQB1*02:02	0.088	0.075	1.16 (0.54–2.52)	0.690	0.828
Combined ACCME and PACS Cohorts					
DRB1*15:03	0.1592	0.2004	0.75 (0.65–0.87)	1.73E-04	0.0017
DRB1*13:02	0.0743	0.0544	1.46 (1.18–1.79)	3.84E-04	0.0019
DRB1*03:01	0.1003	0.0765	1.30 (1.08–1.56)	0.0047	0.0155
DQB1*06:02	0.2405	0.2756	0.84 (0.74–0.95)	0.0078	0.0468
DQB1*02:01	0.141	0.1199	1.20 (1.03–1.41)	0.0195	0.0584
DQB1*02:02	0.0512	0.0668	0.78 (0.61–0.99)	0.0481	0.0962
DQA1*05:01	0.1157	0.0931	1.22 (1.03–1.44)	0.0174	0.1046
B*15:03	0.068	0.0547	1.27 (1.02–1.58)	0.0288	0.1568
B*58:02	0.0414	0.0569	0.74 (0.57–0.97)	0.0349	0.1568
C*06:02	0.0673	0.0832	0.81 (0.65–1.01)	0.0651	0.228
C*07:01	0.1073	0.1283	0.83 (0.70–0.99)	0.0467	0.228

Zygosity tests showed that the heterozygous DRB1*15:03 (OR: 1.25, *p* = 0.01) or DQB1*06:02 (OR: 1.25, *p* = 0.009) allele was associated with a higher risk of persistent hrHPV infections. The heterozygous DRB1*13:02 (OR: 0.68, *p* = 0.002) or DRB1*03:01 (OR: 0.78, *p* = 0.02) allele was associated with a lower risk of persistent hrHPV infections (Supplementary Table 6). Zygosity tests for amino acid residues showed that only homozygous DRB1_59_H and C_48_A were significantly associated with prevalent hrHPV, while 62 heterozygous amino acids were significantly associated with persistent hrHPV (Supplementary Table 7).

### HLA peptide-binding affinity predictions

**Fig. 2:**
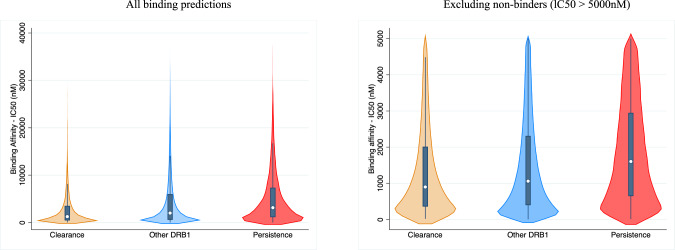
Distribution of binding affinity IC50 values by HLA-DRB1 allele category (**A**) all binding predictions (**B**) Excluding non-binders (lC50 > 5000 nM).

The analyses of peptide binding predictions showed that HLA-DRB1 alleles that were positively associated with persistent hrHPV showed weaker binding with peptides derived from hrHPV proteins and vice versa, as shown by IC50 and EL score data (Fig. 2, Supplementary Table 8). In contrast to other HLA-DRB1 alleles, those alleles positively associated with persistent hrHPV had significantly higher IC50 (and lower EL scores), while negatively associated alleles had significantly lower IC50 (and higher EL scores). These findings remained consistent with consideration of the full set of peptide predictions and after excluding the set of non-binders (IC50 > 5000 nM, Supplementary Table 9). Therefore, persistent alleles were associated with weaker peptide binding and vice versa.

### Gene enrichment analysis

The MAGMA analysis performed with the meta-analyzed data, identified over 100 significant gene sets associated with prevalent and persistent hrHPV. The top gene set associated with prevalent hrHPV was the PID P53 downstream pathway (*p* = 0.001), which includes 134 genes located in CP canonical pathways (Supplementary Table 10). The p53 downstream genes are involved in cell cycle arrest, DNA repair, senescence, apoptosis, and cancer. The antigen processing and presentation gene-set located in CP: KEGG (canonical pathways) of C2 was the top gene-set associated with persistent HPV (*p* = 0.001) (Supplementary Table 11). Antigen presentation is mediated by major histocompatibility complex (MHC) class I.

### Polygenic risk scores

**Fig. 3:**
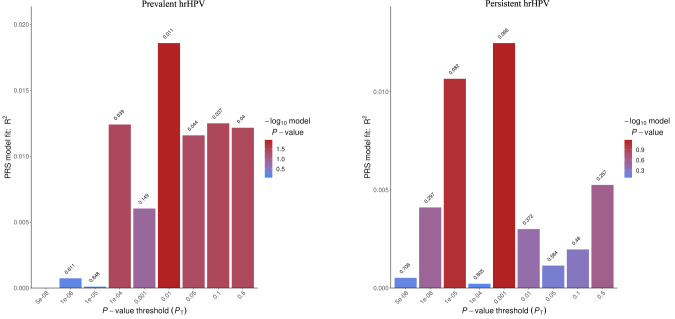
Polygenic Risk Score on predicted risk of prevalent and persistent HPV.

The bar plots displaying the model fit of the PRS at different _PTs_ that illustrate the effect of increasing PRS on the predicted risk of prevalent and persistent hrHPV are shown in Fig. 3. For prevalent hrHPV, restricting SNPs with a more stringent p-value resulted in improvement in the PRS model, with pseudo-R2 of the model reaching 3.3% and *p*-value 0.001 (Table 3). The models were significant for prevalent hrHPV with PRSice-2 and PRS-CS, and borderline significant for persistent hrHPV, with PRS-CS. The results of the two PRS methods had good correlation (0.69) for prevalent hrHPV and modest correlation (0.48) for persistent hrHPV (Supplementary Fig. 8).

**Table 3:** PRS Results Summary.

PRSice-2						
Trait	Threshold	PRS R2	Coefficient	SE	*P*-value	Number of SNPs
Prevalent hrHPV	0.01	0.0193159	322.294	127.243	0.0113124	22441
Prevalent hrHPV	0.00035005	0.0334709	136.304	41.492	0.00101958	1621
Persistent hrHPV	0.001	0.0126097	−183.944	99.9877	0.0658168	3498
Persistent hrHPV	0.00115005	0.0157882	−221.945	108.265	0.0403624	3925
**PRS-CS**						
**Trait**	phi*	**Nagelkerke’s pseudo-R2**	**Estimate**	**SE**	***P*-value**	**Number of SNPs**^**#**^
Prevalent hrHPV	auto	0.01383	0.24078	0.11008	0.02872	1,113,733
Prevalent hrHPV	0.01	0.02581	0.32424	0.10895	0.00292	1,113,733
Persistent hrHPV	auto	0.00059	−0.05354	0.13358	0.68858	1,179,565
Persistent hrHPV	0.01	0.00056	−0.05329	0.13719	0.69767	1,179,565

*global shrinkage parameter.

^#^the number of SNPs in common between the ACCME GWAS, the PRS-CS African LD panel (containing HapMap3 SNPs) and the PACS target dataset.

## Discussion

To our knowledge, this is the first sufficiently powered GWAS of cervical hrHPV infections. Our analyses revealed several loci associated with prevalent and persistent hrHPV infections at genome-wide significance levels. The top variant associated with prevalent hrHPV infection was rs116471799, near *LDB2*. This association was confirmed in the meta-analysis, which also yielded additional variants in and around *PPP3CA* and *NCK2* to be associated with prevalent hrHPV infection. rs2342234 near *TPTE2*, rs115537401 near *SMAD2*, and rs1879062 and rs1028206 clustered around *CDH12* were associated with persistent hrHPV infection. The meta-analysis showed that variants clustered around *TPTE2* were also associated with persistent hrHPV. HLA alleles, haplotypes, and amino acids encoded by DRB1 and DQB1 were strongly associated with persistent hrHPV infection.

### Prevalent HPV

*LDB2*, also known as Carboxyl-Terminal LIM Domain-Binding Protein 1 (*CLIM1*), is a protein coding gene that was identified as an LIM domain-associated cofactor and functions as a transcriptional regulatory factor. *LDB2* is expressed regionally, especially by the uterus and cervix. Previous studies have shown that *LDB2* plays an important role in atherosclerosis development and targets carotid artery disease by inhibiting the activity of transendothelial migration in the leukocyte pathway [12]. Recent in vitro studies showed that *LDB2* inhibited the proliferation and migration of hepatocarcinoma cells and that *LDB2* expression can be upregulated by the m^6^A reader YTH Domain Containing 2 (YTHDC2) in lung adenocarcinoma to inhibit the proliferation of tumor cells [13]. The role of *LDB2* in hrHPV infection remains to be elucidated.

*PPP3CA* is a protein coding gene that is mainly expressed in the brain and prostate. It encodes the catalytic subunit A of calcineurin (or serine/threonine-protein phosphatase 2B catalytic subunit alpha isoform - PP2BA), a calcium- and calmodulin-dependent serine-threonine protein phosphatase that plays critical roles in calcium-dependent signals and T-lymphocyte activation pathways [14]. Mutations in *PPP3CA* may lead to neurodevelopmental disorders and epilepsy [15]. Data from the Human Protein Atlas showed that the protein is expressed in mostly thyroid, prostate and head and neck cancers, while 27% (3/11) of cervical cancer samples showed high/medium expression [16].

*NCK2* encodes a member of the NCK family of adaptor proteins. It shows cytoplasmic and membranous expression in several tissues and is highly expressed by female genital and reproductive tissues, including the cervix, uterus, ovaries, fallopian tubes, breast, and vagina (GTEx). The *NCK2* cytoplasmic protein has been shown to bind and recruit various proteins involved in the regulation of receptor protein tyrosine kinases [17, 18]. It is a biomarker of ovarian cancer progression and a potential target for cancer therapy [19].

### Persistent HPV

*TPTE2* encodes phosphatidylinositol 3,4,5-trisphosphate 3-phosphatase and is a human homologue of *PTEN* (phosphatase and tensin homologue) [20]. *PTEN* is a multifunctional tumor suppressor that is commonly lost in human cancer. The tumor-suppressor activity of *PTEN* depends largely on its lipid phosphatase activity, which opposes Phosphoinositide 3-kinases/Protein Kinase B (PI3K/Akt) activation [21]. Therefore, *PTEN* regulates many cellular processes, including proliferation, survival, energy metabolism, cellular architecture, and motility [21]. Loss of function of *PTEN* can facilitate tumorigenesis and metastasis, and in nontumorigenic cell lines, it can result in phenotypic changes associated with both processes [22]. Overexpressing *TPTE2* in *PTEN*^−/−^ mutant cells reverses all of the phenotypic changes associated with the *PTEN* mutation and in the cases of wound healing and annexin V binding, accentuating the normal *PTEN*^*+/+*^ phenotype [23]. Overexpression of *TPTE2* cDNA in HeLa cells strongly inhibits cell growth/proliferation and causes apoptosis [24]. These findings support our results showing that *TPTE2* is associated with a 65% lower risk of persistent hrHPV infections.

We found a positive association with *SMAD2*, which encodes a SMAD protein. SMAD proteins, such as receptor-regulated SMAD (R-SMAD), are intracellular signal transducers and transcriptional modulators that are activated by transforming growth factor (TGF-β) and activin type 1 receptor kinases [25]. SMAD proteins bind the TRE element in the promoter region of many genes that are regulated by TGF-β and, upon formation of the SMAD2/SMAD4 complex, activate transcription [25]. Disruption of the TGFβ-SMAD signaling pathway due to mutations in SMAD proteins has been described in relation to hrHPV infections of the cervix, cancers of the female reproductive system, including breast cancer, ovarian cancer and cervical cancer, and other cancers. Several studies have reported reduced expression of SMAD2 in human cervical tumor samples [26, 27]. A recent candidate gene study investigated the association between SMAD2 gene polymorphisms and cervical cancer among women in a Bangladeshi population using rs4940086 tagSNP and found that its heterozygous genotype (T/C) was associated with a significantly higher risk of cervical cancer [28]. In our study, we did not find associations with rs4940086, and the frequency of the T/C allele was 0.93/0.06 in Africans, compared to 0.65/0.35 in South Asians.

Cadherin-12 (*CDH12*) is ubiquitously expressed in a wide range of cell types, including the cervix. The CHD12 *g*ene encodes cadherin 12, a member of the cadherin superfamily [29]. Cadherins are homophilic calcium-dependent cell adhesion proteins [30]. Cadherin adhesion molecule is one of the ubiquitous types of cell-cell interactions required for the maintenance of complex tissue morphology [31]. Cadherins play crucial roles during embryonic development and during the maintenance of adult tissues’ normal architecture [32, 33]. Thus, several human diseases result from compromised cadherin expression and function, including cancer [34]. *CDH12* is a subtype of neural cadherin (N-cadherin) [35]. The cadherin switch, defined by N-cadherin expression and characterized by drastic changes in cell polarity, adhesion, and motility, which lead from an E-cadherin-positive differentiated epithelial state into a dedifferentiated mesenchymal-like state prone to metastasis, a development process described in the progression of most epithelial cancers, is a hallmark of epithelial to mesenchymal transition [36]. *CDH12* has been reported to promote proliferation, migration, invasion, adhesion, and angiogenesis, suggesting that it may be an oncogene, diagnostic, and prognostic marker for several cancers [31]. Recent studies have shown a positive association between the expression of N-cadherin, cervical hrHPV infection, and progression of cervical neoplastic lesions [37].

Associations between HLA DRB1 and DQB1 alleles with hrHPV types and cervical cancer have been reported in multiple studies across diverse populations throughout the world; however, the findings have been inconsistent [38–42]. Previous meta-analyses reported that DRB1*13:02 [43, 44] was negatively associated, while DRB1*15:03 [44] was positively associated with cervical cancer. This contrasts with the direction of the association of these alleles in this study. Another meta-analysis reported that DRB1* 03:01 was positively associated with cervical cancer [45], which is similar to our findings. However, the studies included in these meta-analyses had small sample sizes and different methodologies. Our results are supported by a large GWAS using data from 4769 CIN3 and invasive cervical cancer cases and 145,545 controls from UK Biobank. The study reported a significant association with HLA-DQB1, OR = 0.66 [46]. The antigen processing and presentation gene set associated with persistent hrHPV in the present study is mediated by MHC class I and II molecules, which are encoded by HLA genes. Antigen processing and presentation is the first step in the activation of the immune response and a major cellular mechanism through which cells are monitored by the immune system. Failure of immune cells to effectively recognize antigens may contribute to hrHPV persistence. Our HLA peptide-binding prediction analyses show that persistent HLA-DRB1 alleles (HLA-DRB1*03:01 and HLA-DRB1*13:02) were significantly associated with weaker binding with peptides derived from hrHPV proteins in comparison with other alleles. This finding suggests that hrHPV persistence may be associated with a diminished immune response due to weaker binding of hrHPV epitopes, a hypothesis that obtains support from the finding that the “nonpersistence” or “clearance” allele (HLA-DRB1*15:03) displays stronger peptide binding predictions.

This is the first adequately powered GWAS of prevalent and persistent hrHPV infection, and it revealed significant associations with genetic variants, gene sets and pathways, HLA alleles, haplotypes, and amino acids. Our study has several limitations. Although we replicated some of the borderline significant variants, we were unable to replicate the top variants identified in the discovery cohort and confirm all the previous variants associated with HPV in previous studies. This is due to a dearth of data on genomics risk of HPV infections, the small sample size of our replication cohort, variability in the types of HPV and/or population differences (e.g. in allele frequency differences). Although we identified some variants that were statistically significant at the conventional genome-wide threshold, not all of the variants reached the suggested genome-wide threshold for African populations 3.24 × 10^−8^. We were unable to examine the effects of gene-environment interactions with HIV and HPV co-infections because the discovery cohort included only HIV negative women, this may be examined in future studies. Our study focused on group-specific hrHPV infection status, and we did not explore GWAS of type-specific hrHPV infections. Given the very low prevalence of persistent type-specific hrHPV infection, an adequately powered GWAS of persistent type-specific hrHPV infection would require a sample size that is orders of magnitude greater than that in this study.

## Conclusions

The results of this study, which is the second GWAS on hrHPV conducted so far and the first to be adequately powered, show significant loci for prevalent hrHPV infections around *LDB2*, significant loci near *TPTE2, SMAD2*, and *CDH12* for persistent hrHPV infections, and significant polygenic risk scores. Our study revealed that genes in the HLA region, DRB1* and DQB1*, and antigen processing and presentation gene set are statistically significantly associated with persistent hrHPV infections. Further investigations of variations at these loci may provide insight into the mechanisms of susceptibility to hrHPV infection. Larger discovery and replication studies in different populations are warranted to confirm our findings.

## Materials and methods

### Study population

The participants were from the ACCME prospective cohort study of host germline, somatic and HPV genomics and epigenomics, vaginal microenvironment and their associations with persistent high-risk HPV infections and cervical cancer in HIV-negative women. The study eligibility criteria and enrollment procedures have been described in detail elsewhere [11]. The replication cohort included participants in PACS. PACS enrolled women in a study of cervical HPV infection and cervical cancer between 2012 and 2014 at National Hospital, Abuja and University of Abuja Teaching Hospital, Nigeria, as previously described [1, 47–49]. At enrollment in ACCME and PACS, participants consented and underwent the same study procedures, which included the collection of demographic and social data. All the participants were asked to return for follow-up visits, which occurred approximately nine months after baseline, in both cohorts.

### Study endpoints

There were two endpoints in our study: prevalent hrHPV infection and persistent hrHPV infection. We defined infection as prevalent if at least one hrHPV genotype was detected in the baseline sample only despite multiple testing and persistent if at least one hrHPV genotype was detected in samples provided at both the baseline and the 12-month follow-up visit. We defined persistently negative as absence of hrHPV genotype in the baseline and 12-month follow-up visit samples. Participants with persistent hrHPV were not included in the prevalent hrHPV analysis.

### Genotyping and imputation

All samples from participants in the ACCME study were genotyped at the Center for Inherited Disease Research (CIDR) at Johns Hopkins University, using the Illumina H3Africa_2017_20021485 Array. Samples from the replication cohort, PACS, were genotyped either on the Illumina H3Africa_2017_20021485 Array or on the Illumina Multi-Ethnic Global Array (MEGA), at the National Human Genome Research Institute, National Institutes of Health (NIH) and CIDR. Imputation of all samples was performed into the TOPMed reference panel. The resulting imputation dataset of all samples was filtered for variants with MAF ≥ 0.01 and information score (info) ≥ 0.3 for association analysis.

### Case-control genome-wide association analysis

Using LD-pruned SNP genotype data, we computed principal components based on the variance-standardized relationship matrix using PLINK 1.9 using the parameter “--indep 50 5 2”. We determined the number of PCs to include in the association analysis by plotting the scree plots of the PCs (Supplementary Fig. 1) and a Tracy-Widom test. The association between the genetic variants and each study endpoint was estimated using unconditional multivariable logistic regression, assuming an additive genetic model adjusted for age and the first three PCs, with PLINK 1.9. We plotted the qq-plots for the GWASs (Supplementary Fig. 9). The genomic inflation factor was 1.01646 for prevalent high-risk HPV and 1.01583 for persistent high-risk HPV. Genome-wide significance was set at the conventional *p*-value < 5 × 10^−8^. We considered the empirical genome-wide significance threshold of 3.24 × 10^−8^ for African populations. Replication analysis was conducted using the same model, the genome-wide significance was set at *p*-value < 0.05. Further, we conducted a GWAS with a multinomial logistic regression model, with persistent and prevalent hrHPV as case strata and the controls as baseline, using SNPTEST2.

The methods for meta-analysis, evaluation of published HPV and cervical cancer SNPs, functional annotation, Gene Enrichment Analysis, polygenic risk score, classic HLA allele imputation, HLA association analysis, HLA peptide-binding affinity predictions and power calculations are described in the Supplementary Methods.

### Supplementary information

Supplementary methods

Supplementary figures

Supplementary tables

Supplementary table 3

Supplementary table 10

Supplementary table 11

## Data Availability

The datasets used and/or analyzed during the current study available from the corresponding author on reasonable request.
