# Supplementary material for: Genome, HLA and polygenic risk score analyses for prevalent and persistent cervical human papillomavirus (HPV) infections
Source: Eur J Hum Genet. 2024 Jan 10;32(6):708–16. doi: 10.1038/s41431-023-01521-7 (PMC11153215; doi:10.1038/s41431-023-01521-7)
Supplement: Supplementary file 1 — Supplementary methods [file 41431_2023_1521_MOESM1_ESM.pdf]

Genome, HLA and Polygenic Risk Score Analyses for Prevalent and Persistent Cervical Human Papillomavirus (HPV) Infections in HIV-Negative Women

Sally N. Adebamowo[1,2], Adebowale Adeyemo[3], Amos Adebayo[4], Peter Achara[5], Bunmi Alabi[6], Rasheed A. Bakare[7], Ayotunde O. Famooto[8], Kayode Obende[9], Richard Offiong[10], Olayinka Olaniyan[11], Sanni Ologun[12], Charles Rotimi[3], ACCME Research Group as part of the H3Africa Consortium, Clement A. Adebamowo*[1,2,8]

Author Affiliations

[1]Department of Epidemiology and Public Health, University of Maryland School of Medicine, Baltimore, Maryland, 21201, USA

[2]Greenebaum Comprehensive Cancer Center, University of Maryland School of Medicine, Baltimore, Maryland, 21201, USA

[3]National Human Genome Research Institute, Bethesda, USA

[4]Asokoro District Hospital, Abuja, Nigeria

[5]Federal Medical Center, Keffi, Nigeria

[6]Wuse General Hospital, Abuja, Nigeria

[7]Department of Microbiology, University College Hospital, University of Ibadan, Ibadan, Nigeria

[8]Institute of Human Virology Nigeria, Abuja, Nigeria

[9]Garki Hospital Abuja, Abuja, Nigeria

[10]University of Abuja Teaching Hospital, Gwagwalada, Abuja, Nigeria

[11]National Hospital Abuja, Abuja, Nigeria

[12] Kubwa General Hospital Abuja, Nigeria

Supplementary Methods

Meta-analysis

Following the discovery and replication analyses, we conducted a meta-analysis by combining the study-specific GWAS summary statistics for each endpoint in our study in *METAL and* GWAMA.

Evaluation of Published HPV and Cervical Cancer SNPs

We evaluated whether SNPs previously reported to be associated with HPV or cervical cancer in the literature and those that reached suggestive genome-wide significance in our previous GWAS were associated with the study endpoints in the ACCME cohort.

Functional annotation

We used HaploReg to examine the regulatory potentials of the significant variants, and GARFIELD v2 to integrate the GWAS data with regulatory and functional annotations from ENCODE and the Epigenomics Roadmap.

Gene Enrichment Analysis

Gene-based association analysis was conducted with the Multimarker Analysis of GenoMic Annotation (MAGMA). Gene-level analysis was performed with the NCBI 37.3 gene definitions, and the sum of -log(SNP p-value) test statistics was used to evaluate the mean SNP associations.

Polygenic Risk Score

PRS were calculated with PRSice-2 and PRS-CS, using PACS as the target dataset and ACCME as the base dataset. In addition to the standard stringent quality control (QC) steps performed in

the GWAS for both datasets, closely related individuals, SNPs with very high or low heterozygosity rates in individuals The P-value threshold, P_T, with the largest R^2 was the most predictive p-value cut-off. For PRS-CS, the African LD panel was used as the reference.

Classic HLA allele imputation

We used the HLA genotype imputation with attribute bagging (HIBAG) algorithm to derive high-resolution classical HLA types.

HLA Association Analysis

We used PyHLA for the HLA association analysis. We used additive logistic regression models adjusted for age and the first three principal components to test for associations between the four-digit HLA allele and both prevalent and persistent cervical hrHPV infections.

HLA Peptide-binding Affinity Predictions

We analyzed HLA peptide-binding affinity predictions with hrHPV viral proteins. We predicted the peptide-binding affinity of each HLA-DRB1 protein observed with a frequency ≥ 0.01 in our dataset, with all potential 15-mer CD4 epitopes derived from proteins in the UniProt Knowledgebase (KB) and used the NetMHCIIpan 4.1 server for binding affinity predictions. **Supplementary Table 12** shows the list and accession numbers of hrHPV protein sequences retrieved from UniProtKB.

Power Calculations

The power of the discovery case-control study was estimated using the package *genpwr*[50] in R version 4.1.1 (R Core Team 20xx), assuming an α of 5×10^{-8}, under a logistic model. For the prevalent hrHPV analysis, the study had 80% power to detect an odds ratio of 2.0, for a variant with a minor allele frequency (MAF) of 0.18; and 95% power to detect an odds ratio of 2.5, for a variant with MAF of 0.2. Similarly, for the persistent hrHPV analysis, the study had 80% power to detect an odds ratio of 2.0, for a variant with a minor allele frequency (MAF) of 0.05; and 95% power to detect an odds ratio of 1.8, for a variant with MAF of 0.1.
