# Supplementary material for: Genome, HLA and polygenic risk score analyses for prevalent and persistent cervical human papillomavirus (HPV) infections
Source: Eur J Hum Genet. 2024 Jan 10;32(6):708–16. doi: 10.1038/s41431-023-01521-7 (PMC11153215; doi:10.1038/s41431-023-01521-7)

Supplementary Figure 1. Principal component analysis and scree plot for participants in ACCME

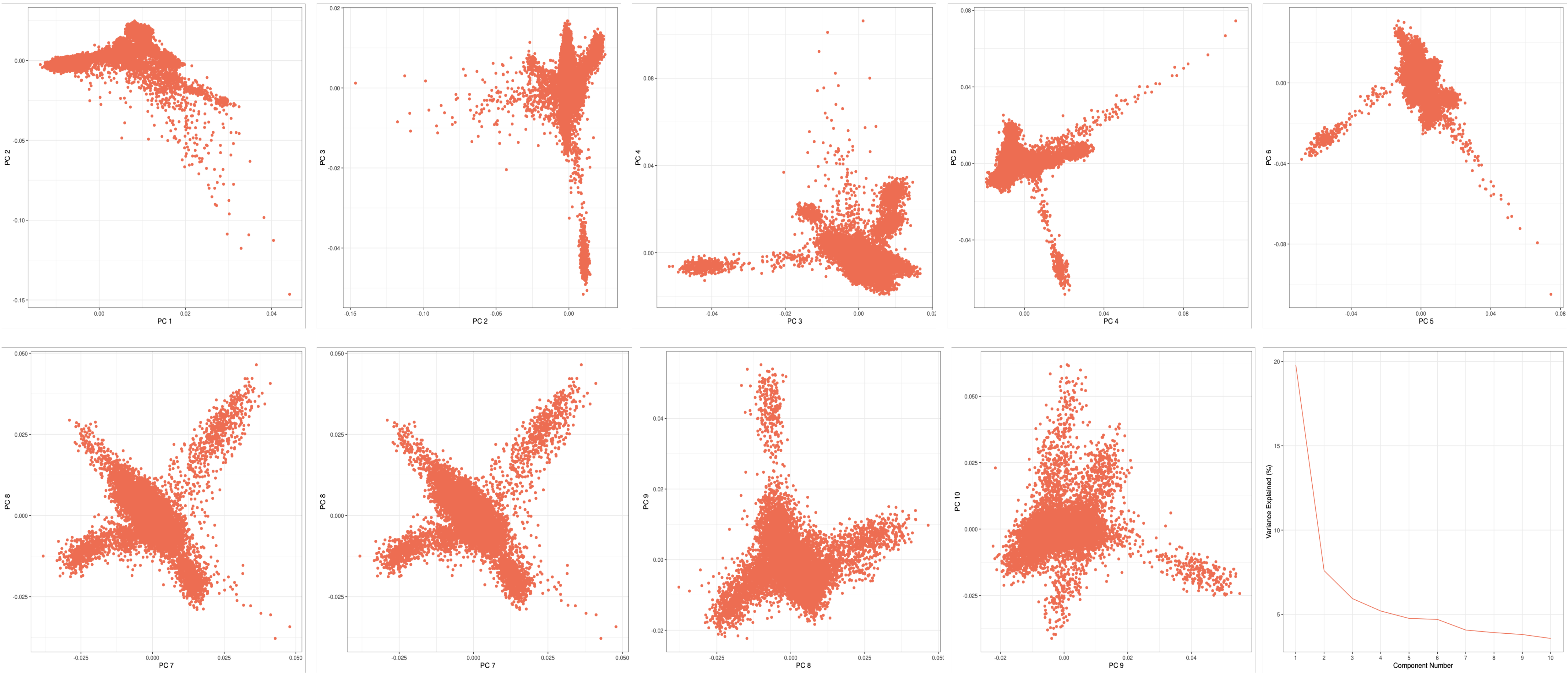

Supplementary Figure 2. Miami Plot for prevalent and persistent hrHPV GWAS

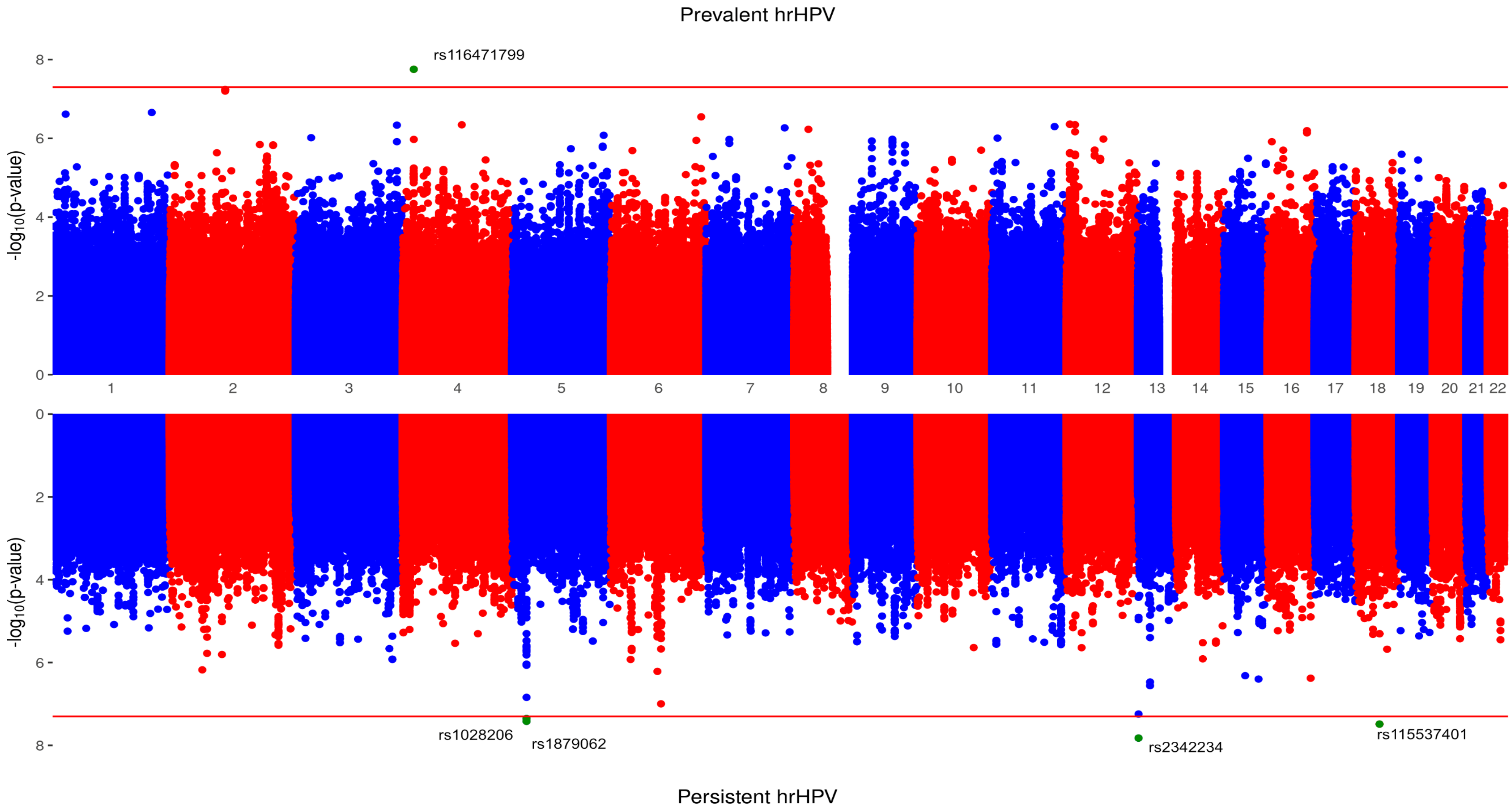

Supplementary Figure 3. Manhattan Plot for multinomial logistic regression GWAS

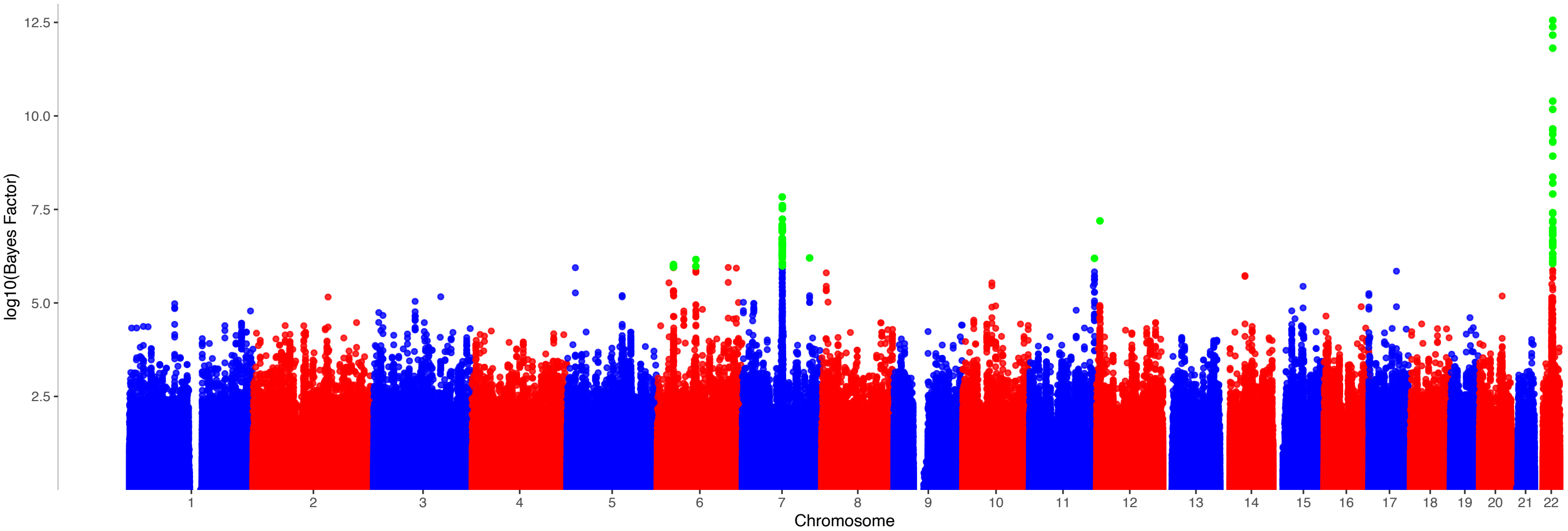

Supplementary Figure 4. Regional plot of the top locus associated with (A) prevalent high-risk HPV (B) persistent high-risk HPV

A

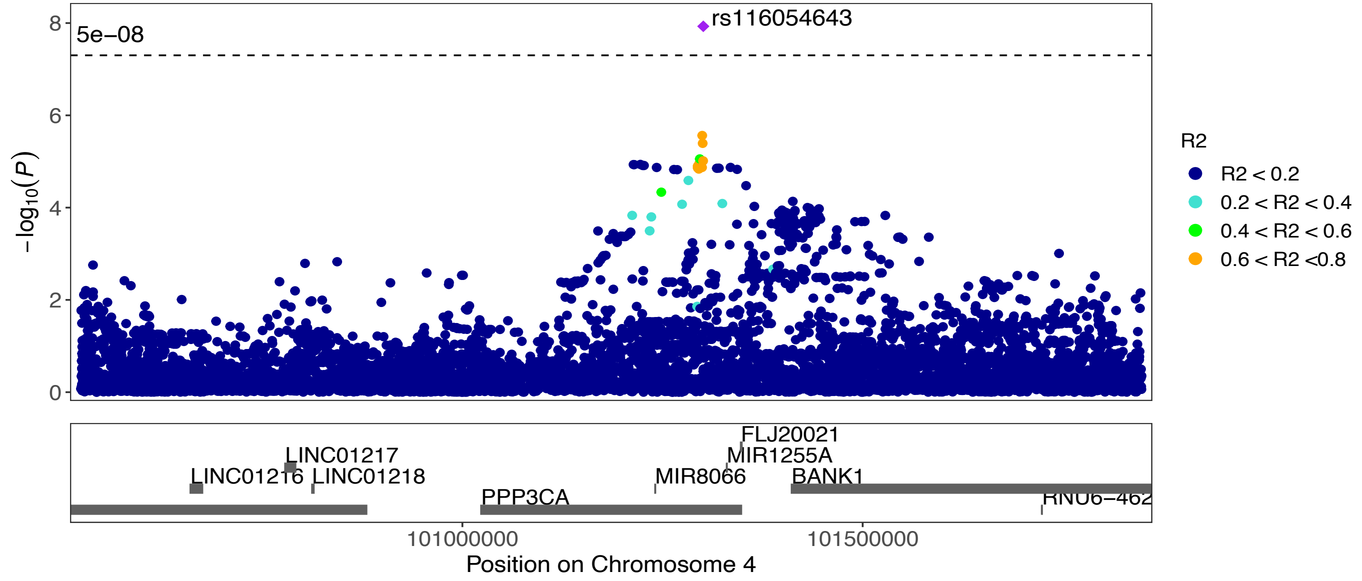

B

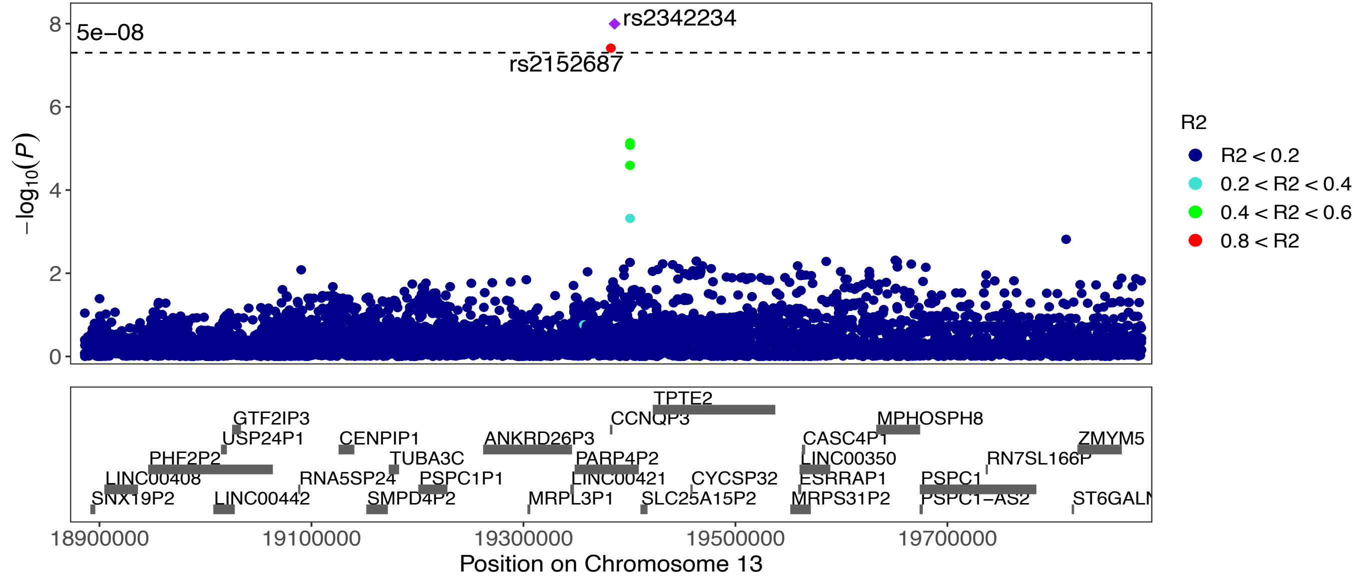

Supplementary Figure 5. Bulk tissue gene expression for LDB2 (ENSG00000169744.12) (Source: GTEx)

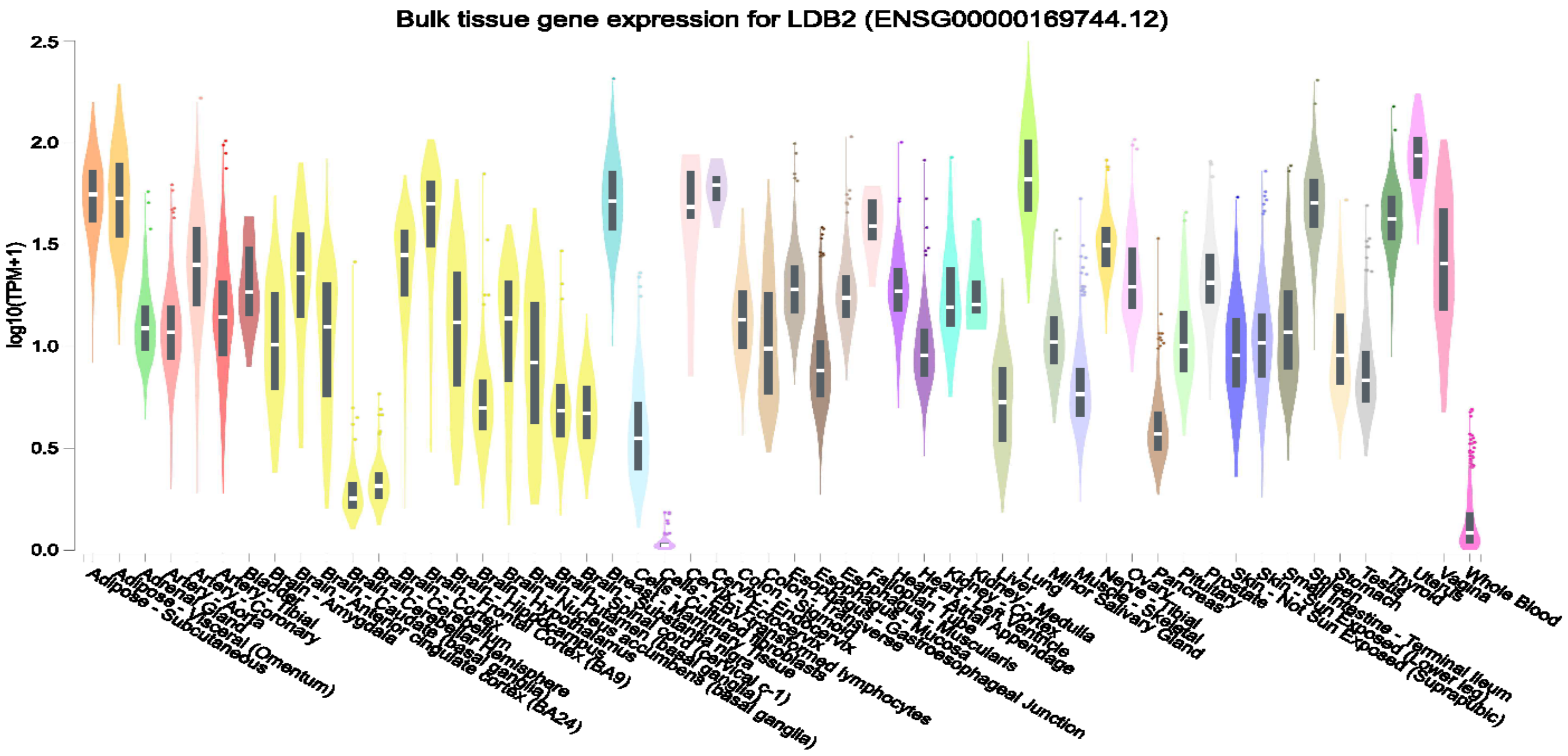

Supplementary Figure 6. Bulk tissue gene expression for TPTE2 (ENSG00000132958.17) (Source: GTEx)

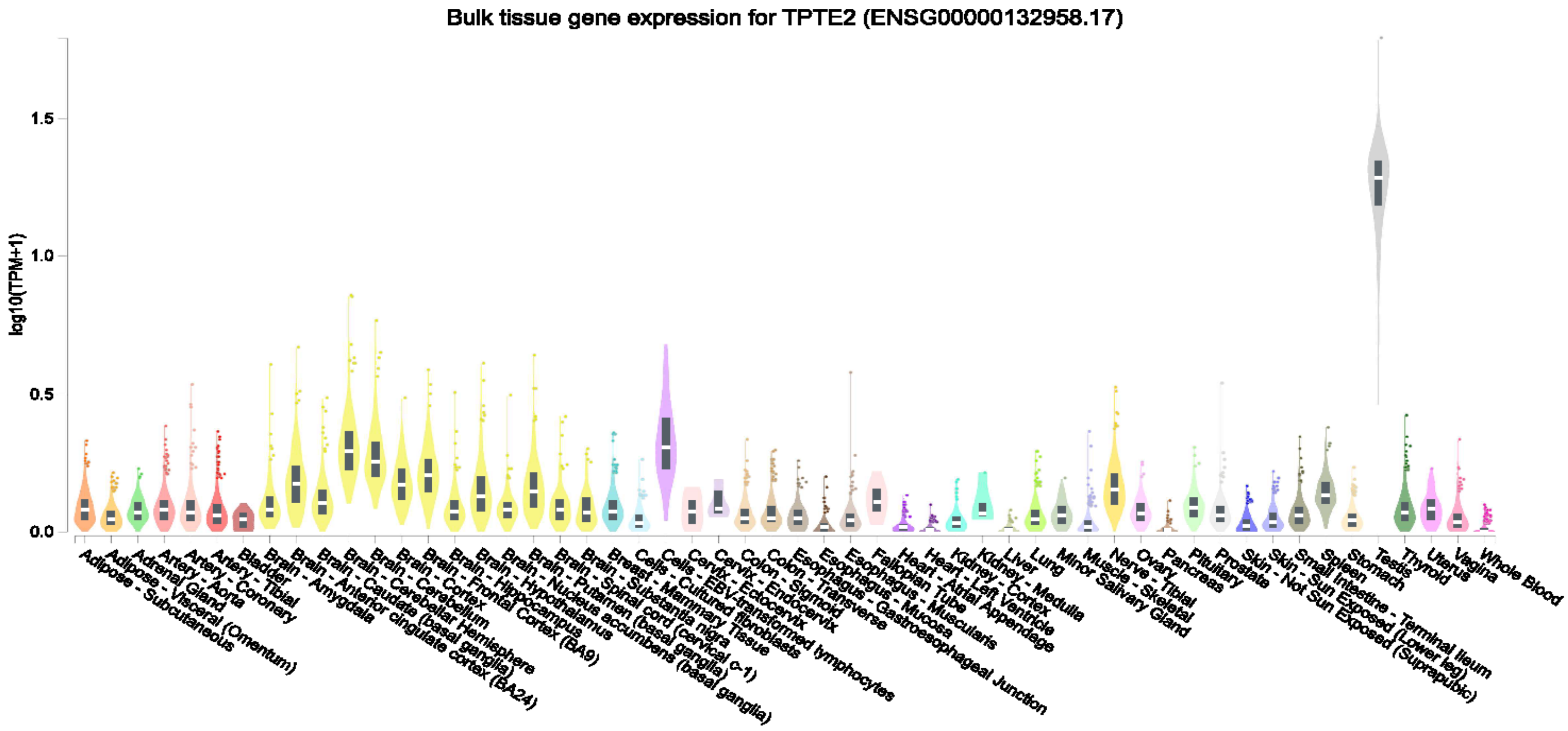

Supplementary Figure 7. Bulk tissue gene expression for SMAD2 (ENSG00000175387.15) (Source: GTEx)

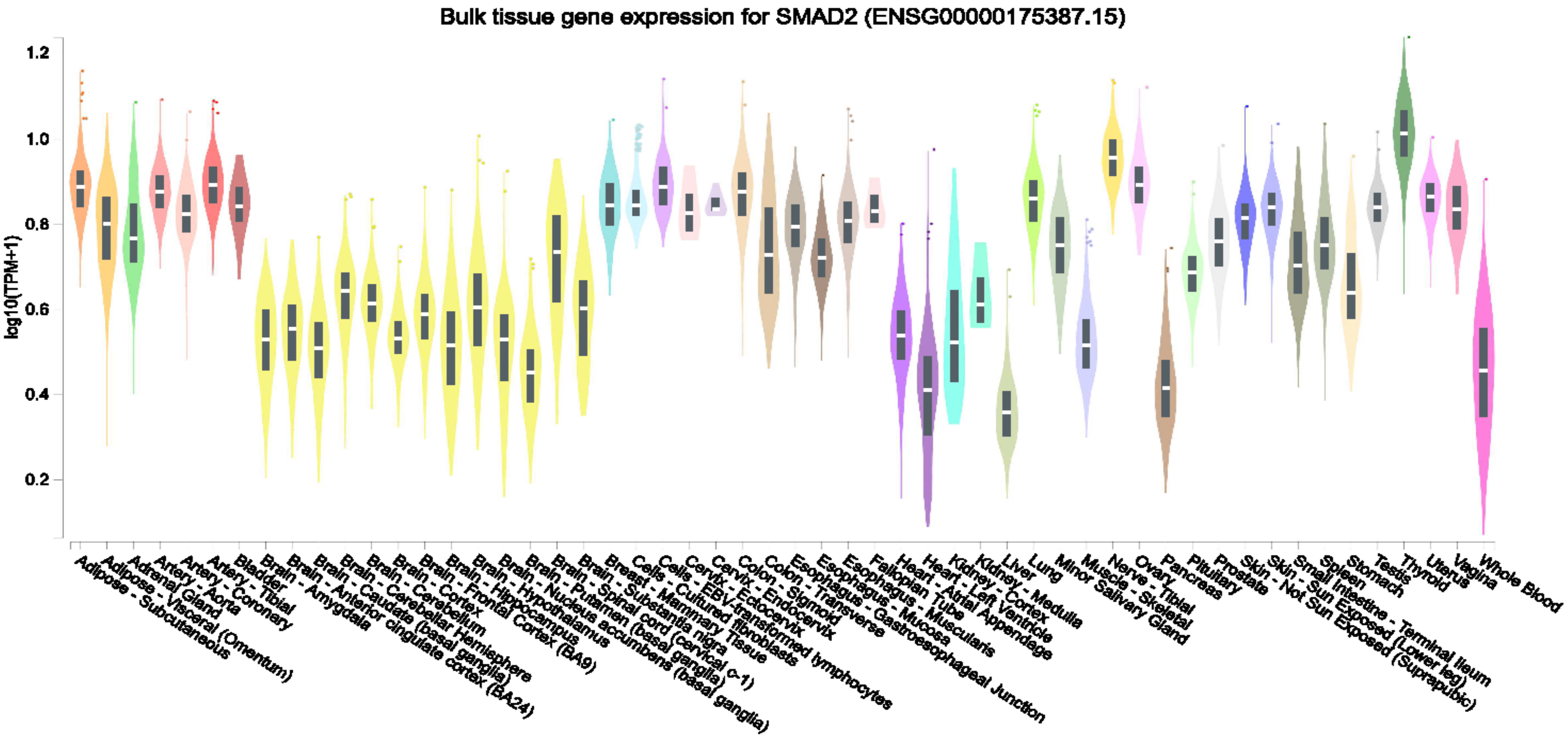

Supplementary Figure 8. Comparison of PRS results with PRSice-2 and PRS-CS

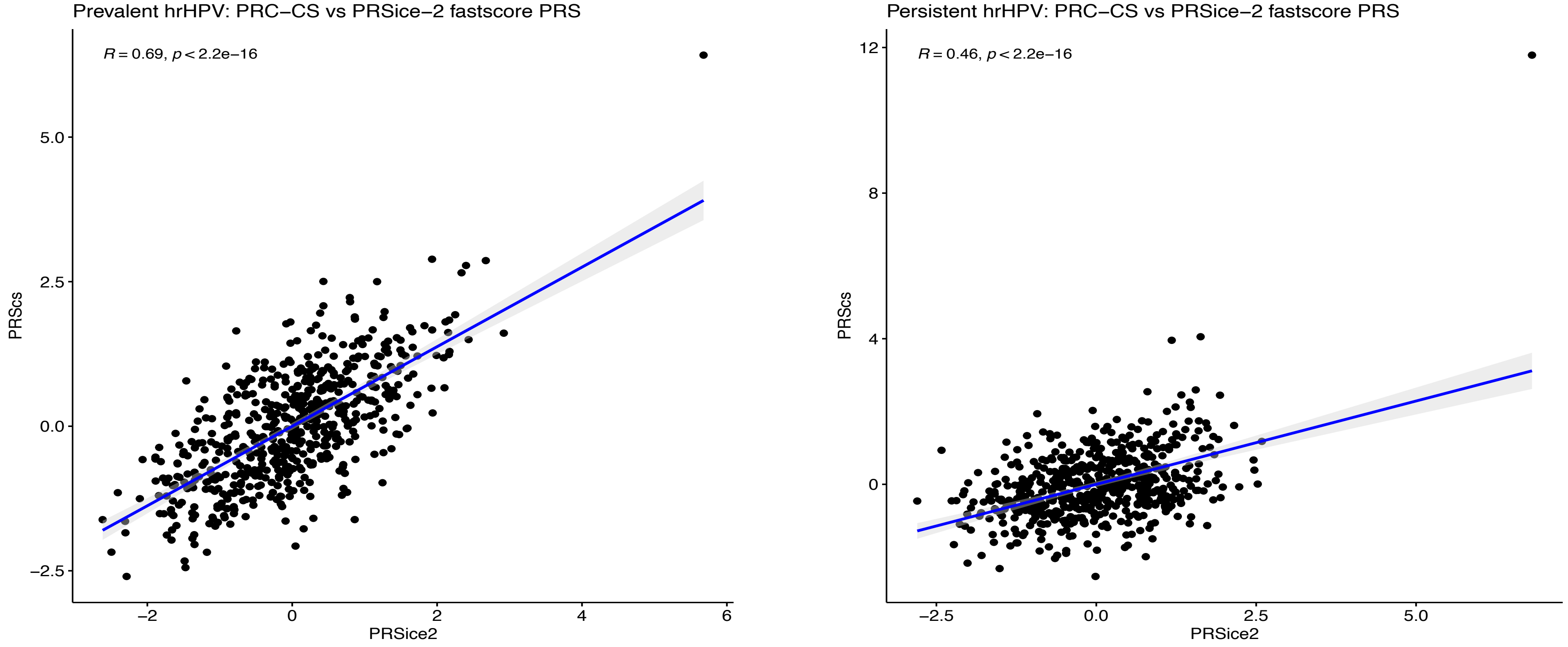

To compare the PRSice-2 results with the PRS-CS results (with phi = 0.01), the obtained PRS scores of were standardized (mean = 0, sd = 1) and Pearson's correlations computed. Scatter plots of the PRSs were generated, showing correlation coefficients and p-values on the plots.

Supplementary Figure 9. QQ-plots for (A) prevalent high-risk HPV (B) persistent high-risk HPV

A

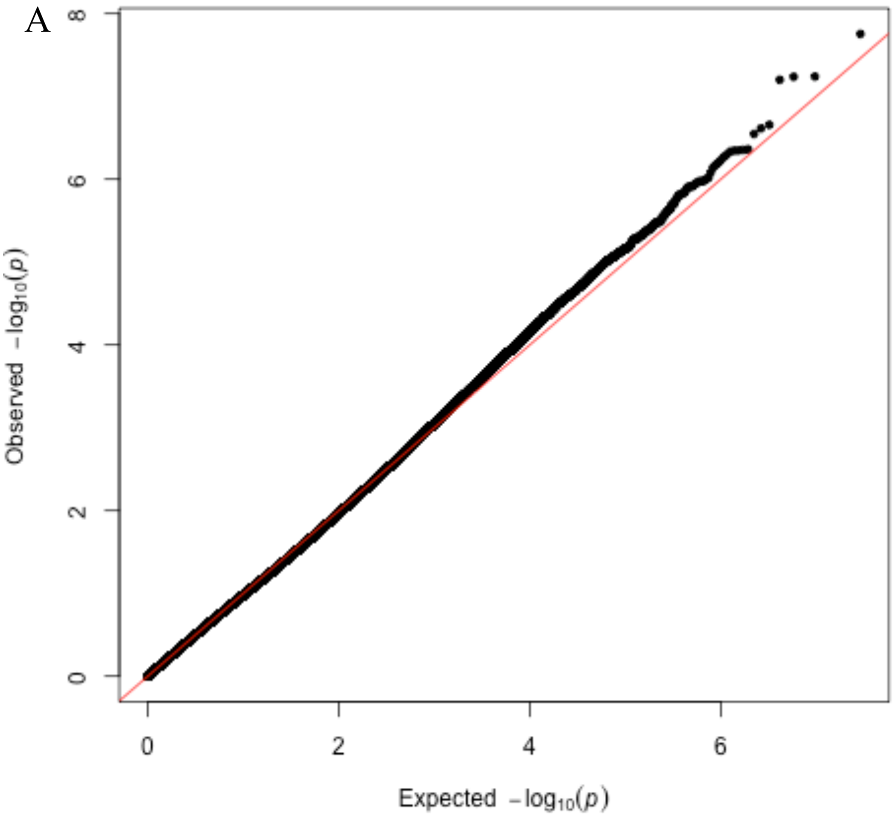

B

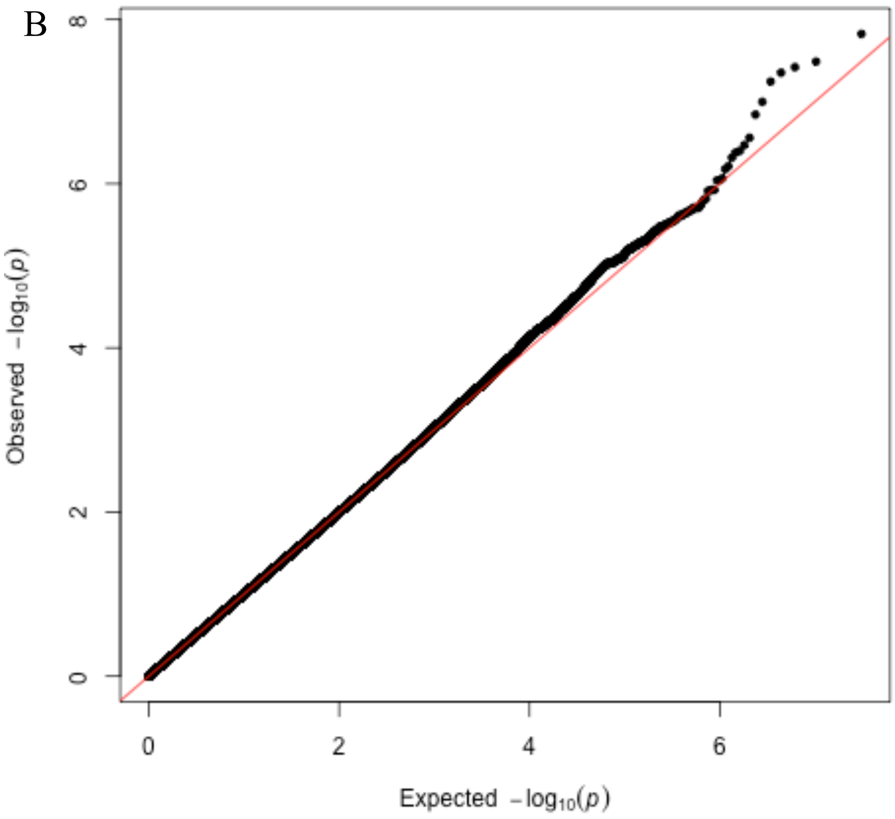

Supplement: Supplementary file 2 — Supplementary figures [file 41431_2023_1521_MOESM2_ESM.pdf]
