# Supplementary material for: Genome, HLA and polygenic risk score analyses for prevalent and persistent cervical human papillomavirus (HPV) infections
Source: Eur J Hum Genet. 2024 Jan 10;32(6):708–16. doi: 10.1038/s41431-023-01521-7 (PMC11153215; doi:10.1038/s41431-023-01521-7)
Supplement: Supplementary file 3 — Supplementary tables [file 41431_2023_1521_MOESM3_ESM.docx]

**Supplementary Tables**

**Genome, HLA and Polygenic Risk Score Analyses for Prevalent and Persistent Cervical Human Papillomavirus (HPV) Infections in HIV-Negative Women**

Sally N. Adebamowo^1,2^, Adebowale Adeyemo^3^, Amos Adebayo^4^, Peter Achara^5^, Bunmi Alabi^6^, Rasheed A. Bakare^7^, Ayotunde O. Famooto^8^, Kayode Obende^9^, Richard Offiong^10^, Olayinka Olaniyan^11^, Sanni Ologun^12^, Charles Rotimi^3^, ACCME Research Group as part of the H3Africa Consortium, Clement A. Adebamowo*^1,2,8^

**Author Affiliations**

^1^Department of Epidemiology and Public Health, University of Maryland School of Medicine, Baltimore, Maryland, 21201, USA

^2^Greenebaum Comprehensive Cancer Center, University of Maryland School of Medicine, Baltimore, Maryland, 21201, USA

^3^National Human Genome Research Institute, Bethesda, USA

^4^Asokoro District Hospital, Abuja, Nigeria

^5^Federal Medical Center, Keffi, Nigeria

^6^Wuse General Hospital, Abuja, Nigeria

^7^Department of Microbiology, University College Hospital, University of Ibadan, Ibadan, Nigeria

^8^Institute of Human Virology Nigeria, Abuja, Nigeria

^9^Garki Hospital Abuja, Abuja, Nigeria

^10^University of Abuja Teaching Hospital, Gwagwalada, Abuja, Nigeria

^11^National Hospital Abuja, Abuja, Nigeria

^12^ Kubwa General Hospital Abuja, Nigeria

| **Supplemental Table 1.** Top Associations with Baseline Prevalent and Persistent Cervical hrHPV Infections | | | | | | | | | | | | |
| --- | --- | --- | --- | --- | --- | --- | --- | --- | --- | --- | --- | --- |
|  |  |  |  |  | Discovery Cohort | | | | Replication Cohort | | | |
| SNP | Chr | Base Position | Near gene | Ref. allele | MAF | OR | SE | *P*-value | MAF | OR | SE | *P*-value |
| **Prevalent hrHPV** | | | | | | | | | | | | |
| rs116471799 | 4 | 16424409 | *LDB2* | C | 0.01 | 3.63 | 0.23 | **1.76 x 10^-8^** | 0.05 | 1.48 | 0.49 | 0.41 |
| rs138289957 | 2 | 105685243 | *NCK2* | A | 0.04 | 2.55 | 0.17 | 5.76 x 10^-8^ | 0.04 | 0.74 | 0.37 | 0.41 |
| rs13407090 | 2 | 105690494 | *NCK2* | A | 0.04 | 2.55 | 0.17 | 5.80 x 10^-8^ | 0.04 | 0.74 | 0.37 | 0.41 |
| rs75543399 | 2 | 105670030 | *NCK2* | A | 0.04 | 2.53 | 0.17 | 6.30 x 10^-8^ | 0.04 | 0.74 | 0.37 | 0.40 |
| rs73099266 | 1 | 217883848 | *SPATA17* | G | 0.10 | 1.92 | 0.13 | 2.20 x 10^-7^ | 0.11 | 1.19 | 0.24 | 0.46 |
| rs4654837 | 1 | 23233507 | *HTR1D* | C | 0.10 | 0.52 | 0.13 | 2.43 x 10^-7^ | 0.11 | 1.40 | 0.25 | 0.18 |
| rs73784569 | 6 | 163291426 | *PACRG* | T | 0.03 | 2.62 | 0.19 | 2.84 x 10^-7^ | 0.03 | 0.59 | 0.46 | 0.25 |
| rs140234575 | 12 | 5704125 | *ANO2* | C | 0.02 | 2.86 | 0.21 | 4.35 x 10^-7^ | 0.02 | 1.49 | 0.42 | 0.34 |
| rs141299627 | 12 | 5706080 | *ANO2* | A | 0.02 | 2.86 | 0.21 | 4.42 x 10^-7^ | 0.02 | 1.49 | 0.42 | 0.34 |
| rs114883051 | 12 | 14534204 | *PLBD1* | C | 0.06 | 2.11 | 0.15 | 4.50 x 10^-7^ | 0.05 | 0.73 | 0.36 | 0.38 |
| rs116054643 | 4 | 101300926 | PPP3CA | T | 0.11 | 1.88 | 0.12 | 4.52 x 10^-7^ | 0.10 | 1.68 | 0.21 | 0.01 |
| rs150001771 | 3 | 188894640 | TPRG1 | A | 0.01 | 3.39 | 0.24 | 4.62 x 10^-7^ | 0.01 | 1.49 | 0.59 | 0.50 |
| **Persistent hrHPV** | | | | | | | | | | | | |
| rs2342234 | 13 | 19385993 | *TPTE2* | C | 0.01 | 0.35 | 0.18 | **1.50 x 10^-8^** | 0.01 | 5.64 | 1.68 | 0.30 |
| rs115537401 | 18 | 47802812 | *SMAD2* | A | 0.01 | 2.50 | 0.17 | **3.26 x 10^-8^** | 0.01 | - | - | - |
| rs1879062 | 5 | 23044423 | *CDH12* | G | 0.05 | 1.79 | 0.10 | **3.81 x 10^-8^** | 0.04 | 0.77 | 0.46 | 0.56 |
| rs1028206 | 5 | 23046022 | *CDH12* | G | 0.05 | 1.78 | 0.10 | **4.45 x 10^-8^** | 0.04 | 0.78 | 0.46 | 0.58 |
| rs2152687 | 13 | 19382475 | *TPTE2* | G | 0.01 | 0.38 | 0.18 | 5.71 x 10^-8^ | 0.01 | 6.59 | 1.77 | 0.28 |
| rs2180716 | 6 | 87974113 | *SPACA1* | A | 0.21 | 1.41 | 0.06 | 1.01 x 10^-7^ | 0.23 | 1.11 | 0.19 | 0.58 |
| rs72750684 | 5 | 23050313 | *CDH12* | A | 0.05 | 1.72 | 0.10 | 1.44 x 10^-7^ | 0.05 | 0.78 | 0.45 | 0.58 |
| rs114789544 | 13 | 38085944 | *TRPC4* | T | 0.04 | 1.80 | 0.11 | 2.76 x 10^-7^ | 0.03 | 1.02 | 0.50 | 0.97 |
| rs115982456 | 13 | 38122581 | *UFM1* | A | 0.04 | 1.78 | 0.11 | 3.39 x 10^-7^ | 0.03 | 1.06 | 0.48 | 0.89 |
| rs188897338 | 15 | 88310779 | *NTRK3* | T | 0.01 | 2.66 | 0.19 | 3.99 x 10^-7^ | 0.01 | - | - | - |
| rs12448674 | 16 | 85476219 | GSE1 | T | 0.48 | 0.75 | 0.06 | 4.18 x 10^-7^ | 0.48 | 0.71 | 0.18 | 0.04 |
| rs143857229 | 15 | 61364786 | RORA | T | 0.02 | 2.16 | 0.15 | 4.81 x 10^-7^ | 0.02 | 1.55 | 0.56 | 0.43 |

| **Supplemental Table 2.** Previously reported SNPs Significantly Associated with Cervical hrHPV Infections in the present study | | | | | | | | |
| --- | --- | --- | --- | --- | --- | --- | --- | --- |
| SNP | Chr | Base Position | Near gene | Ref allele | MAF | OR | SE | P-value |
| **Prevalent** | | | | | | | | |
| rs547159095AT | 3 | 169876466 | *LRRC31* | A | 0.0396 | 1.645 | 0.203 | **0.014** |
| rs79373864 | 11 | 101333016 | *PGR* | T | 0.0261 | 0.241 | 0.586 | **0.015** |
| rs55817252 | 11 | 101308739 | *PGR* | A | 0.0256 | 0.250 | 0.580 | **0.017** |
| rs34832875 | 5 | 173722696 | *BOD1* | T | 0.282 | 0.774 | 0.112 | **0.022** |
| rs12054964 | 5 | 173722706 | *BOD1* | T | 0.282 | 0.774 | 0.112 | **0.022** |
| rs3838783 | 11 | 1283476 | *TOLLIP* | T | 0.6696 | 1.255 | 0.106 | **0.033** |
| **Persistent** | | | | | | | | |
| rs34136369 | 10 | 35894333 | *FZD8* | C | 0.142 | 0.798 | 0.088 | **0.011** |
| rs73010959 | 4 | 180388047 | *TENM3* | G | 0.060 | 0.724 | 0.134 | **0.016** |
| rs73010957 | 4 | 180387927 | *TENM3* | T | 0.060 | 0.724 | 0.134 | **0.016** |
| rs73010973 | 4 | 180389537 | *TENM3* | A | 0.060 | 0.725 | 0.134 | **0.017** |
| rs112893815 | 4 | 180390101 | *TENM3* | T | 0.060 | 0.726 | 0.134 | **0.017** |
| rs74739185 | 4 | 180389866 | *TENM3* | T | 0.060 | 0.725 | 0.134 | **0.017** |
| rs79140020 | 4 | 180389867 | *TENM3* | A | 0.060 | 0.725 | 0.134 | **0.017** |
| rs73010975 | 4 | 180389551 | *TENM3* | A | 0.060 | 0.726 | 0.134 | **0.017** |
| rs73010952 | 4 | 180387229 | *TENM3* | C | 0.060 | 0.730 | 0.134 | **0.019** |
| rs111800742 | 4 | 180387434 | *TENM3* | T | 0.060 | 0.731 | 0.134 | **0.019** |
| rs115749570 | 4 | 3690096 | *ADRA2C* | T | 0.070 | 0.776 | 0.120 | **0.035** |
| rs6731882 | 2 | 15133266 | *NBAS* | T | 0.066 | 1.246 | 0.106 | **0.038** |
| rs74736912 | 4 | 3690072 | *ADRA2C* | T | 0.072 | 0.785 | 0.119 | **0.043** |
| rs72904565 | 2 | 65599788 | *SPRED2* | T | 0.038 | 0.714 | 0.167 | **0.044** |
| rs112931721 | 6 | 33268720 | *VPS52* | G | 0.106 | 0.823 | 0.097 | **0.046** |
| rs11794319 | 9 | 114698942 | *TEX48* | G | 0.127 | 0.838 | 0.089 | **0.047** |
| rs11793753 | 9 | 114697870 | *TEX48* | G | 0.127 | 0.838 | 0.089 | **0.047** |
| rs11794322 | 9 | 114698955 | *TEX48* | G | 0.128 | 0.838 | 0.088 | **0.047** |

| **Supplementary Table 4.** Top 10 HLA Haplotypes Associated with Persistent Cervical hrHPV Infections | | |
| --- | --- | --- |
| Haplotype | OR | FDR Adjusted *P* value |
| C*07:01 - DQB1*06:02 | 0.61 | **2.37 x 10^-04^** |
| B*58:02 - DRB1*15:03 | 0.34 | **5.91 x 10^-04^** |
| C*07:01 - DRB1*15:03 | 0.62 | **6.40 x 10^-04^** |
| DQB1*05:02 - DRB1*13:02 | 1.87 | **7.36 x 10^-04^** |
| C*06:02 - DQB1*06:02 | 0.53 | **8.43 x 10^-04^** |
| A*32:01 - DRB1*15:03 | 2.5 | **0.0011** |
| C*06:02 - DRB1*15:03 | 0.44 | **0.0011** |
| DQB1*06:02 - DRB1*15:03 | 0.75 | **0.0013** |
| B*45:01 - DRB1*03:01 | 2.63 | **0.0022** |
| B*45:01 - DQB1*02:01 | 2.32 | **0.0027** |

| **Supplementary Table 5.** Top 20 Amino Acids Associated with Persistent Cervical hrHPV Infections | | | | | | |
| --- | --- | --- | --- | --- | --- | --- |
|  | Number of Cases | | Number of Controls | | Odds Ratio (OR) | *P* value |
| ID | Carriers | Non-Carriers | ID | Carriers |  |  |
| DRB1_42_S | 539 | 140 | 7144 | 2702 | 1.45 | **8.19 x 10^-05^** |
| DRB1_59_Y | 600 | 79 | 8181 | 1665 | 1.54 | **2.27 x 10^-04^** |
| DRB1_39_Y | 560 | 119 | 7583 | 2263 | 1.4 | **8.88 x 10^-04^** |
| DRB1_40_S | 560 | 119 | 7583 | 2263 | 1.4 | **8.88 x 10^-04^** |
| DRB1_41_T | 560 | 119 | 7583 | 2263 | 1.4 | **8.88 x 10^-04^** |
| DQB1_89_S | 76 | 603 | 758 | 9088 | 1.52 | **1.95 x 10^-03^** |
| DRB1_59_H | 247 | 432 | 4177 | 5669 | 0.78 | **1.96 x 10^-03^** |
| DRB1_38_E | 580 | 99 | 7945 | 1901 | 1.4 | **2.02 x 10^-03^** |
| DRB1_100_A | 204 | 475 | 3533 | 6313 | 0.77 | **2.13 x 10^-03^** |
| DQA1_153_S | 661 | 18 | 9728 | 118 | 0.44 | **3.93 x 10^-03^** |
| A_67_Q | 679 | 0 | 9757 | 89 | 12.47 | **4.16 x 10^-03^** |
| B_86_G | 173 | 506 | 3016 | 6830 | 0.78 | **4.38 x 10^-03^** |
| B_89_R | 173 | 506 | 3016 | 6830 | 0.78 | **4.38 x 10^-03^** |
| B_90_N | 173 | 506 | 3016 | 6830 | 0.78 | **4.38 x 10^-03^** |
| B_91_M | 173 | 506 | 3016 | 6830 | 0.78 | **4.38 x 10^-03^** |
| B_94_S | 173 | 506 | 3016 | 6830 | 0.78 | **4.38 x 10^-03^** |
| DQB1_119_F | 294 | 385 | 4814 | 5032 | 0.8 | **4.82 x 10^-03^** |
| DRB1_39_Q | 360 | 319 | 5772 | 4074 | 0.8 | **4.83 x 10^-03^** |
| DRB1_40_P | 215 | 464 | 3649 | 6197 | 0.79 | **5.09 x 10^-03^** |
| DRB1_42_R | 215 | 464 | 3649 | 6197 | 0.79 | **5.09 x 10^-03^** |

hrHPV = high-risk human papillomavirus; ID = HLA Gene_Position_Amino acid residue; A = Alanine; D = Aspartic acid; E = Glutamic acid; F = Phenylalanine; G = Glycine; H = Histidine; I = Isoleucine; K = Lysine; L = Leucine; M = Methionine; N = Asparagine; P = Proline; Q = Glutamine; R = Arginine; S = Serine; Y = Tyrosine.

| **Supplementary Table 6.** **Zygosity Tests for HLA Alleles Associated with Persistent HPV** | | | | | | |
| --- | --- | --- | --- | --- | --- | --- |
|  | Homozygosity | | Heterozygosity | | Zygosity | |
| Allele | OR | *P* value | OR | *P* value | OR | *P* value |
| A*30:02 | 1.418 | 0.733 | 1.371 | 0.022 | 1.035 | 1.000 |
| A*32:01 | 1.356 | 1.000 | 0.643 | 0.017 | 2.111 | 1.000 |
| B*15:03 | 1.354 | 0.739 | 0.762 | 0.028 | 1.776 | 0.450 |
| B*45:01 | 1.817 | 0.567 | 0.694 | 0.020 | 2.619 | 0.444 |
| B*58:02 | 1.879 | 0.435 | 1.477 | 0.008 | 1.272 | 0.753 |
| C*03:04 | 2.677 | 0.164 | 0.799 | 0.093 | 3.352 | 0.101 |
| C*06:02 | 2.468 | 0.051 | 1.433 | 0.003 | 1.722 | 0.171 |
| C*07:01 | 0.572 | 0.212 | 1.211 | 0.057 | 0.472 | 0.058 |
| DQA1*03:03 | 0.682 | 1.000 | 1.400 | 0.038 | 0.487 | 1.000 |
| DQA1*05:01 | 1.446 | 0.348 | 0.799 | 0.029 | 1.811 | 0.102 |
| DQB1*02:01 | 1.529 | 0.175 | 0.843 | 0.073 | 1.814 | 0.069 |
| DQB1*02:02 | 0.310 | 0.396 | 1.308 | 0.036 | 0.237 | 0.259 |
| DQB1*05:02 | 0.795 | 1.000 | 0.651 | 0.001 | 1.221 | 1.000 |
| DQB1*06:02 | 0.988 | 1.000 | 1.252 | 0.009 | 0.790 | 0.143 |
| DRB1*03:01 | 2.118 | 0.087 | 0.787 | 0.027 | 2.692 | 0.014 |
| DRB1*09:01 | 1.444 | 1.000 | 1.520 | 0.060 | 0.951 | 1.000 |
| DRB1*13:02 | 0.795 | 0.762 | 0.681 | 0.002 | 1.167 | 1.000 |
| DRB1*15:03 | 0.674 | 0.138 | 1.251 | 0.012 | 0.539 | 0.009 |

| **Supplementary Table 7.** **Zygosity Tests for Amino Acid Residues Associated with Persistent HPV** | | | | | | |
| --- | --- | --- | --- | --- | --- | --- |
|  | Homozygosity | | Heterozygosity | | Zygosity | |
| Allele | OR | *P* value | OR | *P* value | OR | *P* value |
| A_67_Q | 0.863 | 0.163 | 0.071 | 0.002 | 12.161 | 0.007 |
| A_89_R | 1.018 | 0.858 | 1.586 | 0.041 | 0.642 | 0.035 |
| A_101_S | 1.356 | 1.000 | 0.643 | 0.017 | 2.111 | 1.000 |
| A_123_Y | 1.018 | 0.858 | 1.586 | 0.041 | 0.642 | 0.035 |
| A_176_R | 1.178 | 1.000 | 1.306 | 0.039 | 0.902 | 1.000 |
| A_180_L | 0.951 | 0.561 | 0.681 | 0.008 | 1.397 | 0.022 |
| A_190_E | 1.037 | 0.690 | 1.642 | 0.020 | 0.631 | 0.024 |
| A_191_W | 1.037 | 0.690 | 1.642 | 0.020 | 0.631 | 0.024 |
| B_2_R | 1.882 | 0.434 | 1.480 | 0.006 | 1.272 | 0.753 |
| B_4_T | 1.882 | 0.434 | 1.480 | 0.006 | 1.272 | 0.753 |
| B_14_W | 1.553 | 0.493 | 1.341 | 0.029 | 1.157 | 1.000 |
| B_15_G | 1.553 | 0.493 | 1.341 | 0.029 | 1.157 | 1.000 |
| B_17_V | 1.553 | 0.493 | 1.341 | 0.029 | 1.157 | 1.000 |
| B_86_G | 0.887 | 0.686 | 1.272 | 0.010 | 0.697 | 0.193 |
| B_89_R | 0.887 | 0.686 | 1.272 | 0.010 | 0.697 | 0.193 |
| B_90_N | 0.887 | 0.686 | 1.272 | 0.010 | 0.697 | 0.193 |
| B_91_M | 0.887 | 0.686 | 1.272 | 0.010 | 0.697 | 0.193 |
| B_93_A | 0.782 | 0.095 | 1.123 | 0.171 | 0.696 | 0.011 |
| B_94_S | 0.887 | 0.686 | 1.272 | 0.010 | 0.697 | 0.193 |
| B_95_A | 0.782 | 0.095 | 1.123 | 0.171 | 0.696 | 0.011 |
| B_101_N | 0.912 | 0.386 | 1.197 | 0.054 | 0.762 | 0.017 |
| B_104_I | 0.895 | 0.359 | 1.151 | 0.106 | 0.778 | 0.037 |
| B_105_A | 0.912 | 0.386 | 1.197 | 0.054 | 0.762 | 0.017 |
| B_106_L | 0.902 | 0.334 | 1.186 | 0.067 | 0.761 | 0.016 |
| B_107_R | 0.902 | 0.334 | 1.186 | 0.067 | 0.761 | 0.016 |
| B_218_V | 1.879 | 0.435 | 1.477 | 0.008 | 1.272 | 0.753 |
| B_269_A | 1.018 | 1.000 | 1.286 | 0.019 | 0.792 | 0.581 |
| B_306_I | 1.880 | 0.435 | 1.479 | 0.006 | 1.272 | 0.753 |
| B_330_T | 1.880 | 0.435 | 1.479 | 0.006 | 1.272 | 0.753 |
| B_350_S | 1.895 | 0.433 | 1.491 | 0.005 | 1.271 | 0.753 |
| C_33_D | 0.804 | 0.176 | 1.184 | 0.043 | 0.680 | 0.012 |
| C_33_Y | 1.045 | 0.696 | 0.797 | 0.012 | 1.310 | 0.026 |
| C_48_A | 1.184 | 0.043 | 0.817 | 0.210 | 1.450 | 0.014 |
| C_48_S | 0.801 | 0.176 | 1.188 | 0.039 | 0.675 | 0.010 |
| C_97_T | 1.001 | 1.000 | 0.800 | 0.009 | 1.252 | 0.097 |
| C_101_N | 1.066 | 0.490 | 1.268 | 0.024 | 0.841 | 0.129 |
| C_104_K | 1.066 | 0.490 | 1.268 | 0.024 | 0.841 | 0.129 |
| C_114_A | 1.030 | 0.790 | 0.803 | 0.017 | 1.283 | 0.036 |
| DQA1_49_S | 1.689 | 0.657 | 1.419 | 0.017 | 1.190 | 1.000 |
| DQA1_70_Q | 1.689 | 0.657 | 1.419 | 0.017 | 1.190 | 1.000 |
| DQA1_73_L | 0.954 | 1.000 | 1.242 | 0.033 | 0.768 | 0.409 |
| DQA1_76_R | 0.954 | 1.000 | 1.242 | 0.033 | 0.768 | 0.409 |
| DQA1_79_R | 1.689 | 0.657 | 1.419 | 0.017 | 1.190 | 1.000 |
| DQA1_99_V | 1.689 | 0.657 | 1.419 | 0.017 | 1.190 | 1.000 |
| DQA1_153_S | 0.904 | 0.313 | 2.117 | 0.010 | 0.427 | 0.004 |
| DQA1_183_D | 0.682 | 1.000 | 1.400 | 0.038 | 0.487 | 1.000 |
| DQA1_210_T | 0.682 | 1.000 | 1.400 | 0.038 | 0.487 | 1.000 |
| DQA1_238_L | 0.756 | 0.488 | 1.200 | 0.081 | 0.630 | 0.214 |
| DQB1_41_F | 1.013 | 0.946 | 1.250 | 0.008 | 0.810 | 0.115 |
| DQB1_46_M | 0.946 | 0.544 | 1.396 | 0.068 | 0.678 | 0.027 |
| DQB1_89_S | 0.795 | 1.000 | 0.651 | 0.001 | 1.221 | 1.000 |
| DQB1_119_F | 1.005 | 1.000 | 1.251 | 0.008 | 0.803 | 0.156 |
| DQB1_119_Y | 1.095 | 0.657 | 0.848 | 0.050 | 1.292 | 0.141 |
| DQB1_167_G | 0.310 | 0.396 | 1.308 | 0.036 | 0.237 | 0.259 |
| DQB1_172_A | 0.310 | 0.396 | 1.308 | 0.036 | 0.237 | 0.259 |
| DQB1_199_R | 0.310 | 0.396 | 1.308 | 0.036 | 0.237 | 0.259 |
| DQB1_214_S | 0.310 | 0.396 | 1.308 | 0.036 | 0.237 | 0.259 |
| DQB1_217_T | 0.310 | 0.396 | 1.308 | 0.036 | 0.237 | 0.259 |
| DRB1_38_E | 1.031 | 0.758 | 0.724 | 0.005 | 1.423 | 0.004 |
| DRB1_38_K | 1.444 | 1.000 | 1.520 | 0.060 | 0.951 | 1.000 |
| DRB1_38_W | 0.856 | 0.293 | 1.154 | 0.088 | 0.742 | 0.035 |
| DRB1_39_Q | 0.822 | 0.155 | 1.203 | 0.029 | 0.684 | 0.004 |
| DRB1_39_Y | 1.019 | 0.855 | 0.719 | 0.002 | 1.418 | 0.003 |
| DRB1_40_D | 1.444 | 1.000 | 1.520 | 0.060 | 0.951 | 1.000 |
| DRB1_40_P | 0.783 | 0.285 | 1.233 | 0.017 | 0.635 | 0.037 |
| DRB1_40_S | 1.019 | 0.855 | 0.719 | 0.002 | 1.418 | 0.003 |
| DRB1_41_K | 0.777 | 0.051 | 1.154 | 0.090 | 0.673 | 0.002 |
| DRB1_41_T | 1.019 | 0.855 | 0.719 | 0.002 | 1.418 | 0.003 |
| DRB1_42_R | 0.783 | 0.285 | 1.233 | 0.017 | 0.635 | 0.037 |
| DRB1_42_S | 1.016 | 0.886 | 0.691 | 0.000 | 1.469 | 0.001 |
| DRB1_57_H | 1.444 | 1.000 | 1.520 | 0.060 | 0.951 | 1.000 |
| DRB1_59_G | 1.444 | 1.000 | 1.520 | 0.060 | 0.951 | 1.000 |
| DRB1_59_H | 0.586 | 0.014 | 1.208 | 0.025 | 0.486 | 0.000 |
| DRB1_59_Y | 1.126 | 0.173 | 0.686 | 0.003 | 1.643 | 0.000 |
| DRB1_61_H | 1.188 | 0.172 | 0.850 | 0.059 | 1.397 | 0.009 |
| DRB1_61_Y | 0.850 | 0.059 | 1.188 | 0.172 | 0.716 | 0.009 |
| DRB1_66_N | 1.215 | 0.168 | 0.843 | 0.043 | 1.442 | 0.010 |
| DRB1_66_S | 0.940 | 0.736 | 1.171 | 0.061 | 0.803 | 0.189 |
| DRB1_86_V | 0.605 | 0.235 | 1.176 | 0.110 | 0.514 | 0.070 |
| DRB1_89_S | 0.605 | 0.235 | 1.176 | 0.110 | 0.514 | 0.070 |
| DRB1_96_I | 0.825 | 0.051 | 1.129 | 0.220 | 0.731 | 0.006 |
| DRB1_96_L | 1.059 | 0.746 | 0.805 | 0.010 | 1.316 | 0.102 |
| DRB1_100_A | 0.737 | 0.218 | 1.258 | 0.010 | 0.586 | 0.019 |
| DRB1_103_R | 1.391 | 0.206 | 0.831 | 0.046 | 1.674 | 0.051 |
| DRB1_106_N | 1.391 | 0.206 | 0.831 | 0.046 | 1.674 | 0.051 |

**Supplementary Table** **8**: HLA-binding affinity IC50 (nM) and Elution score (EL) by HLA-DRB1 allele category

|  | **Median** | **25^th^ percentile** | **75^th^ percentile** |
| --- | --- | --- | --- |
| **Percentile IC50 (nM)** **by HLA-DRB1 allele category** | | | |
| ***All predictions*** |  |  |  |
| Persistence | 3142.525 | 1132.025 | 7369.62 |
| Other | 2006.63 | 618.71 | 5997.65 |
| Non-persistence | 1259.125 | 455.855 | 3527.72 |
| Kruskal-Wallis H | X^2^= 5393.086 (p=0.0001) |  |  |
| ***Excluding non-binders (IC50 > 5000nM)*** | |  |  |
| Persistence | 1603.92 | 646.79 | 2948.95 |
| Other | 1060.895 | 396.825 | 2310.18 |
| Non-persistence | 899.565 | 358.95 | 2010.16 |
| Kruskal-Wallis H (p) | X^2^= 3184.953  (p=0.0001) |  |  |
|  | | | |
| **Percentile Elution score (EL) by HLA-DRB1 allele category** | | | |
| ***All predictions*** |  |  |  |
| Persistence | .0077295 | .002036 | .0316965 |
| Other | .0085025 | .002189 | .0367115 |
| Non-persistence | .0116125 | .003413 | .04324 |
| Kruskal-Wallis H | X^2^= 819.402 (p=0.0001) |  |  |

**Supplementary Table 9**: Multinomial logit regression modeling of the relationship between HLA-DRB1 allele category and (A) binding affinity log IC50 (nM) (B) elution score

(A) binding affinity log IC50 (nM)

| **Category** | **Coefficient (B)** | **SE(B)** | **z** | **P>\|z\|** | **95% CI** |
| --- | --- | --- | --- | --- | --- |
| **All predictions** | | | | | |
| Persistence | 0.393 | 0.007 | 54.25 | <0.001 | 0.379, 0.407 |
| Clearance | -0.351 | 0.009 | -39.13 | <0.001 | -0.368, -0.333 |
| Other alleles (reference) |  |  |  |  |  |
| Model LR chi2(2df) = 4996.61, p<0.0001 | | | | | |
|  | | | | | |
| Excluding non-binders (IC50 > 5000 nM) | | | | | |
| Persistence | 0.593 | .0120 | 49.51 | <0.001 | 0.570, 0.616 |
| Clearance | -0.134 | .0126 | -10.63 | <0.001 | -0.159, -0.109 |
| Other alleles (reference) |  |  |  |  |  |
| Model LR chi2(2df) = 2893.95, p< 0.0001 | | | | | |

(B) Elution score

| **Category** | **Coefficient (B)** | **SE(B)** | **z** | **P>\|z\|** | **95% CI** |
| --- | --- | --- | --- | --- | --- |
| Persistence | -0.556 | .046 | -12.20 | <0.001 | -0.645, -0.466 |
| Clearance | 0.135 | .054 | 2.50 | 0.013 | 0.029, 0.242 |
| Other alleles (reference) |  |  |  |  |  |
| Model LR chi2(2df) = 172.14, p < 0.0001 | | | | | |

**Supplementary Table 12**: List and accession numbers of hrHPV protein sequences retrieved from UniProtKB

| **Proteome ID** | **Organism** | **Organism ID** |
| --- | --- | --- |
| UP000009109 | Human papillomavirus type 18 | 333761 |
| UP000009251 | Human papillomavirus type 16 | 333760 |
| UP000125839 | Human papillomavirus 59 | 37115 |
| UP000007668 | Human papillomavirus 58 | 10598 |
| UP000007666 | Human papillomavirus 56 | 10596 |
| UP000008692 | Human papillomavirus 52 | 10618 |
| UP000096939 | Human papillomavirus 45 | 10593 |
| UP000113298 | Human papillomavirus 35 | 10587 |
| UP000009118 | Human papillomavirus 33 | 10586 |
| UP000009116 | Human papillomavirus 31 | 10585 |
| UP000156231 | Human papillomavirus 39 | 10588 |
| UP000129881 | Human papillomavirus 51 | 10595 |
